# Online Digital Education for Postregistration Training of Medical Doctors: Systematic Review by the Digital Health Education Collaboration

**DOI:** 10.2196/13269

**Published:** 2019-02-25

**Authors:** Pradeep Paul George, Olena Zhabenko, Bhone Myint Kyaw, Panagiotis Antoniou, Pawel Posadzki, Nakul Saxena, Monika Semwal, Lorainne Tudor Car, Nabil Zary, Craig Lockwood, Josip Car

**Affiliations:** 1 Health Services and Outcomes Research National Healthcare Group Singapore Singapore; 2 Joanna Briggs Institute University of Adelaide Adelaide Australia; 3 Lee Kong Chian School of Medicine Nanyang Technological University Singapore Singapore; 4 Centre for Population Health Sciences Lee Kong Chian School of Medicine Nanyang Technological University Singapore Singapore; 5 Family Medicine and Primary Care Lee Kong Chian School of Medicine Nanyang Technological University Singapore Singapore; 6 Laboratory of Medical Physics Aristotle University of Thessaloniki Thessaloníki Greece; 7 Ophthalmology Team Novartis Singapore Singapore; 8 Medical Education Research and Scholarship Unit Lee Kong Chian School of Medicine Nanyang Technological University Singapore Singapore; 9 Department of Learning, Informative, Management and Ethics Karolinska Institutet Stockholm Sweden; 10 10I Emerging Technologies Lab Mohammed VI University of Health Sciences Casablanca Morocco; 11 Department of Family Medicine Faculty of Medicine University of Ljubljana Ljubljana Slovenia

**Keywords:** randomized controlled trials, effectiveness, systematic review, medical education, internet

## Abstract

**Background:**

Globally, online and local area network–based (LAN) digital education (ODE) has grown in popularity. Blended learning is used by ODE along with traditional learning. Studies have shown the increasing potential of these technologies in training medical doctors; however, the evidence for its effectiveness and cost-effectiveness is unclear.

**Objective:**

This systematic review evaluated the effectiveness of online and LAN-based ODE in improving practicing medical doctors’ knowledge, skills, attitude, satisfaction (primary outcomes), practice or behavior change, patient outcomes, and cost-effectiveness (secondary outcomes).

**Methods:**

We searched seven electronic databased for randomized controlled trials, cluster-randomized trials, and quasi-randomized trials from January 1990 to March 2017. Two review authors independently extracted data and assessed the risk of bias. We have presented the findings narratively. We mainly compared ODE with self-directed/face-to-face learning and blended learning with self-directed/face-to-face learning.

**Results:**

A total of 93 studies (N=16,895) were included, of which 76 compared ODE (including blended) and self-directed/face-to-face learning. Overall, the effect of ODE (including blended) on postintervention knowledge, skills, attitude, satisfaction, practice or behavior change, and patient outcomes was inconsistent and ranged mostly from no difference between the groups to higher postintervention score in the intervention group (small to large effect size, very low to low quality evidence). Twenty-one studies reported higher knowledge scores (small to large effect size and very low quality) for the intervention, while 20 studies reported no difference in knowledge between the groups. Seven studies reported higher skill score in the intervention (large effect size and low quality), while 13 studies reported no difference in the skill scores between the groups. One study reported a higher attitude score for the intervention (very low quality), while four studies reported no difference in the attitude score between the groups. Four studies reported higher postintervention physician satisfaction with the intervention (large effect size and low quality), while six studies reported no difference in satisfaction between the groups. Eight studies reported higher postintervention practice or behavior change for the ODE group (small to moderate effect size and low quality), while five studies reported no difference in practice or behavior change between the groups. One study reported higher improvement in patient outcome, while three others reported no difference in patient outcome between the groups. None of the included studies reported any unintended/adverse effects or cost-effectiveness of the interventions.

**Conclusions:**

Empiric evidence showed that ODE and blended learning may be equivalent to self-directed/face-to-face learning for training practicing physicians. Few other studies demonstrated that ODE and blended learning may significantly improve learning outcomes compared to self-directed/face-to-face learning. The quality of the evidence in these studies was found to be very low for knowledge. Further high-quality randomized controlled trials are required to confirm these findings.

## Introduction

Information communication technology (ICT) has transformed the way information is exchanged and shared around the world [1-4]. In medical education, ICT facilitated a paradigm shift from traditional learning to a dynamic system, moving away from the instructor- or student-focused presentation session to a student-centered process, where students can learn anywhere, anytime, and at their own pace. It also provides unique opportunities for interactive communication and networking [5].

Online and local area network–based (LAN) digital education (ODE) encompasses a variety of interventions characterized by their tools, content, learning objectives, pedagogical approaches, and delivery settings. ODE also varies widely in its configuration (eg, tutorial, asynchronous discussion, and live conferencing), instructional methods (eg, practice exercises and cognitive interactivity), and presentation [6]. ODE uses a full electronic approach, which is entirely driven by technology, or a mix of traditional learning and digital technology (ie, blended learning). Blended learning may be more suitable for health care training, which commonly needs to combine hands-on skill-based training at a practical level and self-directed learning such as ODE [7-9].

ODE has been used widely in undergraduate medical and other health professionals’ education [10] and is now gaining popularity in postregistration medical education for lifelong learning (ie, continuing education), evidenced by the growing number of studies. Continuing medical education (CME) is defined as “all educational activities which serve to maintain, develop, or increase the knowledge, skills, and professional performance and relationships that a physician used to provide services for patients, the public, or the profession” [11] and continuing professional development (CPD) is defined as “a range of learning activities through which medical professionals maintained and developed throughout their career to ensure that they retain their capacity to practice safely, effectively and legally within their evolving scope of practice” [12]. Recently, nearly all medical schools in the United States and Canada moved to providing some form of online learning material as part of their CME for physicians [6].

Research shows that learning is influenced more by the content and instructional strategy than by the type of technology used to deliver the content [13]; in other words, the design of a course determines its effectiveness in learning [14]. There is a significant methodological, educational, and clinical heterogeneity amongst the studies [15-37], which highlighted the need for a review on ODE that focused specifically on the education of medical doctors with more homogenous learning technologies. The *a priori* protocol reported here has also been published in the Cochrane library [38].

The primary objective of this review was to evaluate the effectiveness of ODE in improving doctors’ knowledge, skills, attitude, and satisfaction. The secondary objectives were to assess changes in clinical practices or behaviors, patient outcomes, costs and cost-effectiveness of the intervention, and unintended/adverse effects on patients and physicians.

## Methods

### Search Strategy and Data Sources

A search strategy was developed in accordance with the Cochrane Handbook of Systematic Reviews of Interventions [39] to search the Cochrane CENTRAL, MEDLINE (Ovid), Embase (Elsevier), PsycINFO (Ovid), ERIC (Ovid), CINAHL (Ebsco), Web of Science Core Collection (Thomson Reuters), and International Clinical Trials Platform (World Health Organization) databases. The detailed search strategy is presented in Multimedia Appendix 1, and a detailed description of the methodology has been published elsewhere [40]. Databases were searched from January 1, 1990, to March 9, 2017. We selected 1990 as the starting year for our search because prior to this, the use of ICT for education was limited. We identified additional studies both by scanning relevant systematic reviews and meta-analyses and hand searching reference lists of all included studies.

### Selection Criteria

Only randomized controlled trials (RCTs), cluster RCTs (cRCTs), and quasi-randomized controlled trials of postregistration education for medical doctors using ODE (standalone or blended) with any type of controls measuring knowledge, cognitive skills, attitudes, satisfaction (primary outcome), changes in practice or behavior, patient outcomes, costs, or adverse effects (secondary outcome) outcomes were eligible for inclusion in this review (Multimedia Appendix 2). Participants were not excluded on the basis of age, gender, or any other sociodemographic variables. We used the gold-standard systematic review methods recommended by the Cochrane Collaboration and strictly adhered to the published protocol [38].

### Data Extraction

Three reviewers (PG, OZ, and BK) independently screened the titles and abstracts and full-text versions of the eligible studies and performed the data extraction. We extracted the relevant data on participants, intervention and control, outcome measures, and study designs. We contacted the study authors in cases of any missing information. A fourth review author (PP) acted as an arbiter in cases of disagreement.

### Risk of Bias Assessment

Two reviewers independently assessed the risk of bias for RCTs using the Cochrane Collaboration’s “risk of bias” tool [39]. For RCTs, we did so across the domains of random sequence generation, allocation concealment, blinding of outcome assessment, incomplete outcome data, selective outcome reporting, and other bias including the comparability of intervention and control group; characteristics at baseline; validity and reliability of outcome assessment tools; and protection against contamination. Blinding of participants and personnel was not assessed, as the nature of the intervention precludes blinding.

We assessed the risk of bias for cRCTs across the domains of recruitment bias [41], baseline imbalances, loss of clusters incorrect analysis, and comparability with individual randomized trials [39]. For each study, two reviewers independently categorized each domain as low, high, or unclear risk of bias.

### Assessment of Heterogeneity

Clinical heterogeneity was assessed to check if the included studies were similar in terms of their population, intervention characteristics, and reported outcomes and to ascertain the possibility of pooling the measures of effect. The extracted data were analyzed using RevMan 5.3 software (The Nordic Cochrane Centre, Copenhagen, Denmark). Statistical heterogeneity was assessed using the Chi-square and I^2^ tests [39]. We found significant heterogeneity (clinical and statistical) among the included studies; hence, meta-analysis was not suitable for analysis.

### Data Synthesis

The results from individual RCTs were reported as the standardized mean difference (SMD) for continuous variables and risk ratios (RR) for dichotomous variables. Where studies reported more than one measure for each outcome, the primary measure, as defined by the study authors, was used in the analysis. If studies had multiple arms, we compared the intervention arm to the least active control arm and assessed the difference in postintervention outcomes. Similarly, when multiple domains of the same outcome were measured, only the primary domains identified and agreed upon by the review authors were reported. Meta-analyses were not possible because there was significant clinical and methodological heterogeneity across the included studies.

### Summary of Findings

Summary of findings tables (Tables 1-3) were prepared based on the methods described in Chapter 11 of the Cochrane Handbook for Systematic Reviews of Interventions [39]. Two review authors (PG and BK) independently used the Grading of Recommendations, Assessment, Development and Evaluations (GRADE) criteria to rank the quality of the evidence using the GRADE profiler (GRADEpro) software [42]. In the main review, we only compared ODE with self-directed/face-to-face learning and blended learning with self-directed/face-to-face learning; the rest of the comparisons are presented in Multimedia Appendix 3.

**Table 1:** Summary of findings for online and local area network–based digital education as compared to self-directed learning. patient or population: postregistration medical doctors; setting: universities, hospitals, and primary care; intervention: online and local area network–based digital education; comparison: self-directed learning.

Outcomes	Number of participants (number of RCTs^a^)	Quality of evidence (GRADE^b^)	Direction of effects
Knowledge assessed with multiple-choice questions. Follow-up ranged from posttest to 1 year	3067 (29)	Very low^c,d,e,f^	Seventeen studies [43-60] reported that ODE^g^ was significantly more effective than self-directed learning (very low certainty evidence). Two studies [61,62] reported mixed results (very low certainty evidence). Ten studies [63-72] reported that ODE was as effective as self-directed learning (very low certainty evidence).
Skills assessed with OSCE^h^, diagnostic assessment, examination, questionnaires, and surveys. Follow-up ranged from posttest to 4 years	829 (8)	Low^c,d,i^	Five studies [65,73-76] reported that ODE was significantly more effective than self-directed learning (low certainty evidence). Two studies [77,78] reported that ODE was as effective as self-directed learning (low certainty evidence). One study [54] reported self-directed learning was more effective than ODE (low certainty evidence).
Attitude assessed with questionnaires. Follow-up ranged from posttest to 136 days	392 (4)	Low^c,d^	One study [47] reported that ODE was significantly more effective than self-directed learning (low certainty evidence). Another [66] reported that ODE was as effective as self-directed learning (low certainty evidence). Two studies [44,58] reported mixed results (low certainty evidence).
Satisfaction assessed with questionnaires. Follow-up ranged from posttest to 6 months	934 (6)	Low^c,d^	Two studies [67,79] reported that ODE was significantly more effective (low certainty evidence). Three studies [54,58,80] reported that ODE was as effective as self-directed learning (low-certainty evidence). One study [61] reported mixed results (low certainty evidence).

^a^RCT: randomized controlled trial.

^b^GRADE: Grading of Recommendations, Assessment, Development and Evaluations.

^c^Rated down by one level for study limitations. Most studies were considered to be at an unclear or high risk of bias. Overall, the risk of bias for most studies was unclear due to a lack of information reported.

^d^Rated down by one level for inconsistency. There was variation in effect size (ie, very large and very small effects were observed).

^e^Rated down by one level for publication bias. The effect estimates were asymmetrical, suggesting possible publication bias.

^f^Very low quality (+ – – –): We have very little confidence in the effect estimate. The true effect is likely to be substantially different from the estimate of effect.

^g^ODE: online and local area network–based digital education.

^h^OSCE: objective structured clinical examination.

^i^Low quality (+ + – –): Our confidence in the effect estimate is limited. The true effect may be substantially different from the estimate of the effect

**Table 2:** Summary of findings for online digital education as compared to face-to-face learning. patient or population: postregistration medical doctors; setting: universities, hospitals, and primary care; intervention: online and local area network–based digital education; comparison: face-to-face learning.

Outcomes	Number of participants (number of RCTs^a^)	Quality of evidence (GRADE^b^)	Direction of effects
Knowledge assessed with multiple-choice questions. Follow-up ranged from posttest to 18 months	1202 (9)	Very low^c,d,e,f^	Two studies [81,82] reported that ODE^g^ was significantly more effective in improving physicians’ knowledge scores than face-to-face learning (very low certainty evidence). Six studies [83-88] found that ODE was as effective as face-to-face learning in improving physicians’ knowledge scores (very low certainty evidence). One study [89] reported that face-to-face learning was significantly more effective than ODE in improving physicians’ knowledge scores.
Skills assessed with OSCE^h^, diagnostic assessment, examination, questionnaires, and surveys. Follow-up ranged from posttest to 12 months	291 (7)	Low^c,d,i^	Six studies [84,87,90-93] reported ODE was as effective as face-to-face learning in improving physicians’ skills (low certainty evidence). In one study [94], data were missing.
Attitude assessed with questionnaires. Follow-up ranged from posttest to 18 months	220 (2)	Low^c,d^	Two studies [82,95] reported that ODE was as effective as face-to-face learning in improving physicians’ attitude (low certainty evidence).
Satisfaction assessed with questionnaires. Follow-up ranged from posttest to 12 weeks	260 (4)	Low^c,d^	Two studies [83,87] reported that ODE was significantly more effective than face-to-face learning for improving physicians’ satisfaction (low certainty evidence). Two studies [81,84] reported that ODE was as effective as face-to-face learning in improving physicians’ satisfaction (low certainty evidence).

^a^RCT: randomized controlled trial.

^b^GRADE: Grading of Recommendations, Assessment, Development and Evaluations.

^c^Rated down by one level for study limitations. Most studies were considered to be at an unclear or high risk of bias. Overall, the risk of bias for most studies was unclear due to a lack of information reported.

^d^Rated down by one level for inconsistency. There was variation in effect size (ie, very large and very small effects were observed).

^e^Rated down by one level for publication bias. The effect estimates were asymmetrical, suggesting possible publication bias.

^f^Very low quality (+ – – –): We have very little confidence in the effect estimate. The true effect is likely to be substantially different from the estimate of effect.

^g^ODE: online and local area network–based digital education.

^h^OSCE: objective structured clinical examination.

^i^Low quality (+ + – –): Our confidence in the effect estimate is limited. The true effect may be substantially different from the estimate of the effect

**Table 3:** Summary of findings for blended learning as compared to self-directed/face-to-face learning. patient or population: postregistration medical doctors; setting: universities, hospitals, and primary care; intervention: blended learning; comparison: self-directed/face-to-face learning.

Outcomes	Number of participants (number of studies)	Quality of evidence (GRADE^a^)	Direction of effects
Knowledge assessed with multiple-choice questions. Follow-up ranged from posttest to 26 months	4413 (7 RCTs^b^)	Very low^c,d,e,f^	Two studies [96,97] reported that blended learning was significantly more effective in improving physicians’ knowledge than self-directed/face-to-face learning (very low certainty evidence). Five studies assessed together [96,98-101] reported that blended learning was as effective as self-directed/face-to-face learning (very low certainty evidence).
Skills assessed with OSCE^g^, diagnostic assessment, examination, questionnaires, and surveys. Follow-up ranged from posttest to 26 months.	4131 (6 RCTs)	Low^c,d,h^	Two studies [96,102] reported that blended learning may significantly improve physicians’ skills, and four studies [98,99,103,104] reported that blended learning may be as effective as face-to-face learning in improving skills (low certainty evidence).
Attitude assessed with a questionnaire. Follow-up assessed posttest	61 (1 cRCT^i^)	Low^c,d^	Kulier et al [105] compared a blended learning course on EBM^j^ to a face-to-face EBM course and reported that the intervention may be as effective as the controls for improving physicians’ attitude.
Satisfaction assessed with questionnaires on a Likert scale. Follow-up ranged from posttest to 6 months	166 (3 RCTs)	Low^c,d^	Ali et al [98] compared ATLS^k^ delivered through blended learning to a standard ATLS course and reported no difference in satisfaction between the groups (low certainty evidence). Kronick et al [106] compared 3 hours of online training to no training (self-directed training) and found that the intervention slightly improved satisfaction (low certainty evidence). Platz et al [100] compared basic ultrasound principles and extended focused assessment with sonography for trauma using blended learning as compared to face-to-face training and reported mixed results (low certainty evidence).

^a^GRADE: Grading of Recommendations, Assessment, Development and Evaluations.

^b^RCT: randomized controlled trial.

^c^Rated down by one level for study limitations. Most studies were considered to be at an unclear or high risk of bias. Overall, the risk of bias for most studies was unclear due to a lack of information reported.

^d^Rated down by one level for inconsistency. There was variation in effect size (ie, very large and very small effects were observed).

^e^Rated down by one level for publication bias. The effect estimates were asymmetrical, suggesting possible publication bias.

^f^Very low quality (+ – – –): We have very little confidence in the effect estimate. The true effect is likely to be substantially different from the estimate of effect.

^g^OSCE: objective structured clinical examination.

^h^Low quality (+ + – –): Our confidence in the effect estimate is limited. The true effect may be substantially different from the estimate of the effect.

^i^cRCT: cluster-randomized trial.

^j^EBM: evidence-based medicine.

^k^ATLS: Advanced Trauma Life Support

## Results 

### Search Results

**Figure 1:**
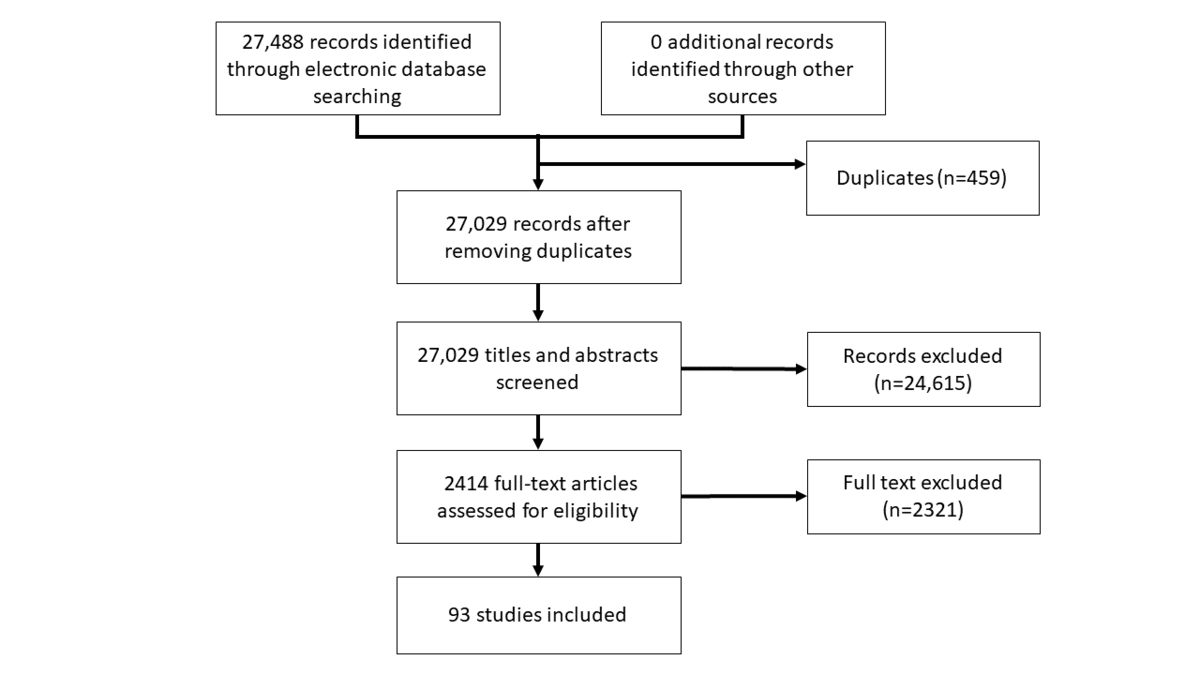
Modified Preferred Reporting Items for Systematic Reviews and Meta-Analyses (PRISMA) flow chart of the search results and study-selection process.

Our searches yielded a total of 27,488 citations and 93 studies (Figure 1). Of those, 74 studies were RCTs including 12,537 participants and 19 were cRCTs including 1262 clusters, 3727 physicians, and 7690 patients. Sixty-four studies were published between 2010 and 2017, and the remaining 29 studies were published between 1999 and 2009.

### Participants, Settings, and Countries of Origin

**Figure 2:**
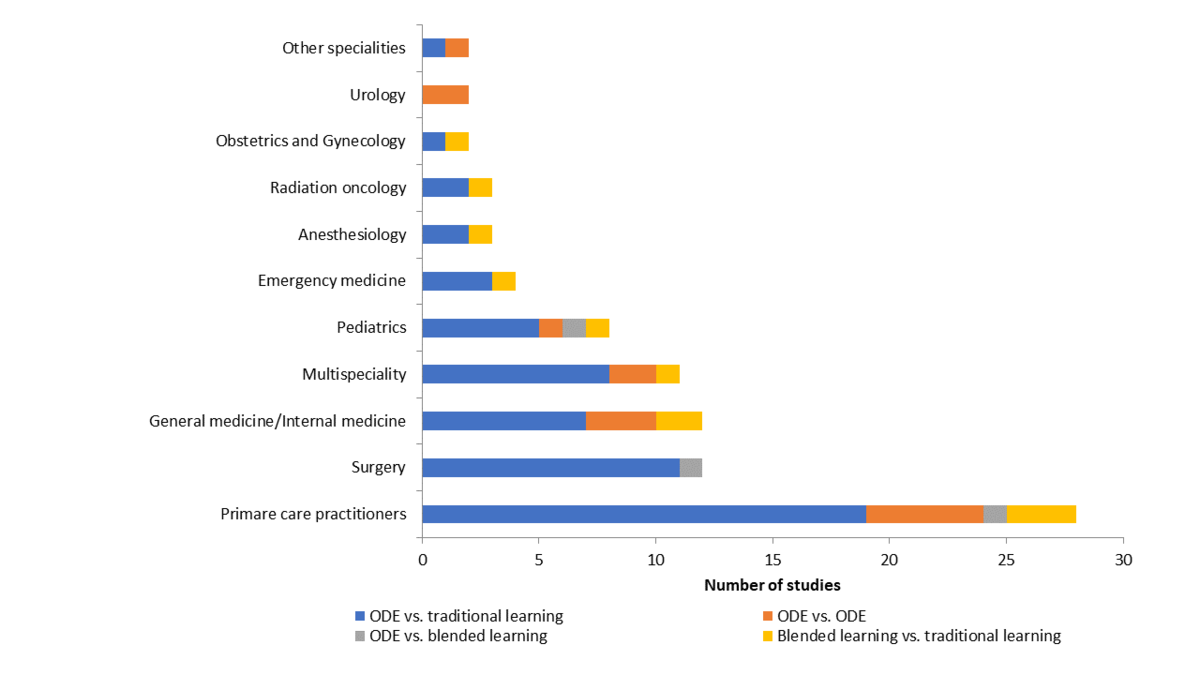
Number of ODE studies by specialty and type of learning. ODE: online and local area network–based digital education.

A total of 29 (31.1%) studies were conducted among primary care practitioners (general practitioners, family medicine practitioners/residents, and occupational physicians), 12 among surgeons (12.9%), 11 among general and internal medicine practitioners (11.8%), and 8 among pediatricians (8.6%; Figure 2 and Multimedia Appendices 4-10). Only 2 (2.2%) [83,96] of the 93 studies were conducted in low- to middle-income countries; all the remaining studies were conducted in high-income countries with majority in the United States (53.8%), Canada (10.8%), and Germany (5.4%; Multimedia Appendix 11). Fifty studies were carried out in hospital settings, 31 studies were conducted in university settings, 11 studies were conducted in primary care settings, and one study was conducted in a mixed hospital and university setting.

### Interventions and Comparisons

A total of 61 studies compared ODE and self-directed/face-to-face learning. Self-directed learning was defined as self-learning through books and journals, and face-to-face learning was defined as learning through didactic classroom lectures and courses. Fourteen studies compared ODE and other types of ODE, 3 studies compared ODE and blended learning, and 15 studies compared blended learning and self-directed/face-to-face learning. Two studies used synchronous learning technology (video-conferencing systems) for training, and 39 studies used asynchronous learning technologies such as Web-based libraries/repositories of video modules, CD-ROM, emails, and online discussion groups to deliver the intervention. In the main review, we only compared ODE with self-directed/face-to-face learning and blended learning with self-directed/face-to-face learning; the rest of the comparisons are presented in Multimedia Appendix 3.

### Risk of Bias in Randomized Controlled Trials and Cluster Randomized Controlled Trials

**Figure 3:**
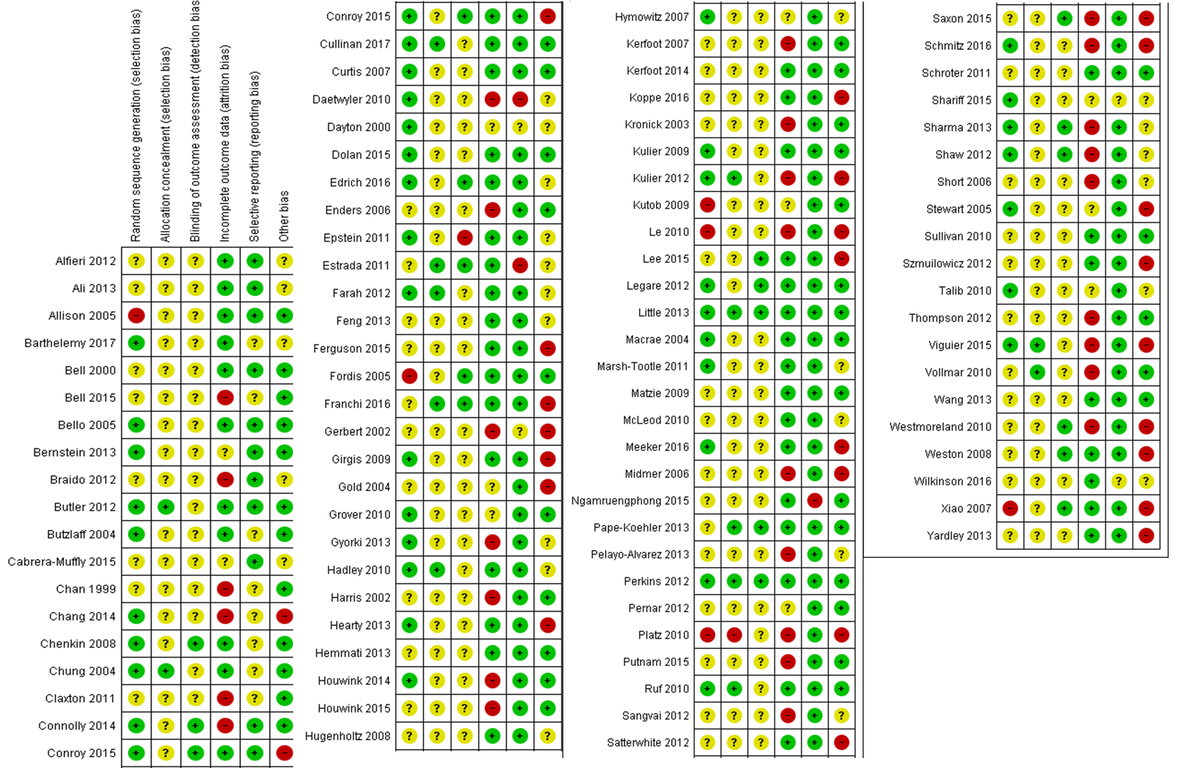
Risk-of-bias summary for each included study.

**Figure 4:**
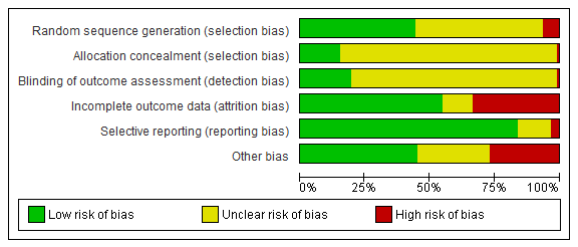
Risk-of-bias item results presented as percentages across all included studies.

A total of 51 studies were considered to be at high risk of bias for at least one of the risk of bias domains (Figures 3 and 4). Six studies were rated as having a high risk of selection bias, 31 studies were rated as having a high risk of attrition bias due to a high drop-out rate (>20%), and 3 studies [103,107,108] had a high risk of reporting bias. Epstein et al reported a high risk of detection bias [109]. Further, 25 studies had a high risk of “other biases.” Similarly, among the cRCTs, 12 studies had a high risk of bias for baseline imbalance, 8 studies had a high risk of bias for loss of clusters, and 3 studies had a high risk of bias for incorrect analyses. Risk of bias is described in detail in Multimedia Appendices 4 and 12.

### Effects of Interventions by Outcomes

The characteristics of included studies categorized by participants’ specialty, outcomes, comparisons, and intervention types are presented in Multimedia Appendices 5-13. The educational content was heterogeneous among the included studies. Studies that compared ODE/blended ODE with self-directed or face-to-face learning are presented in the manuscript; for other comparisons, see Multimedia Appendix 3.

### Primary Outcomes

#### Knowledge

A total of 54 studies assessed knowledge: 20 studies used questionnaires (open ended), 28 studies used multiple-choice questions, and 6 studies did not specify the type of instrument used to measure knowledge.

##### Online and Local Area Network–Based Digital Education Versus Self-Directed Learning

**Figure 5:**
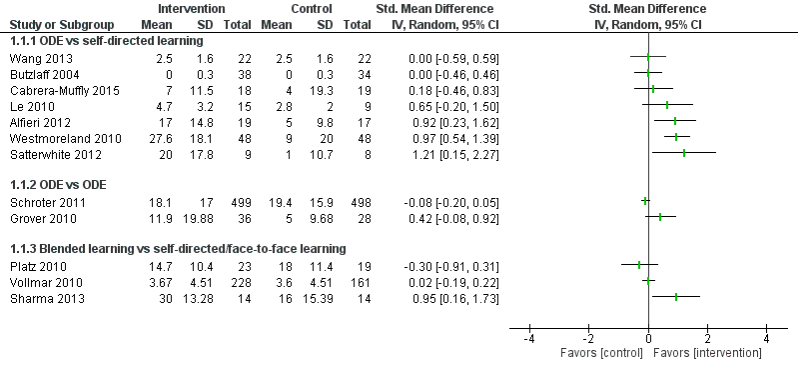
Comparison of change in knowledge scores (postintervention). ODE: online and local area network–based digital education; IV: inverse variance.

**Figure 6:**
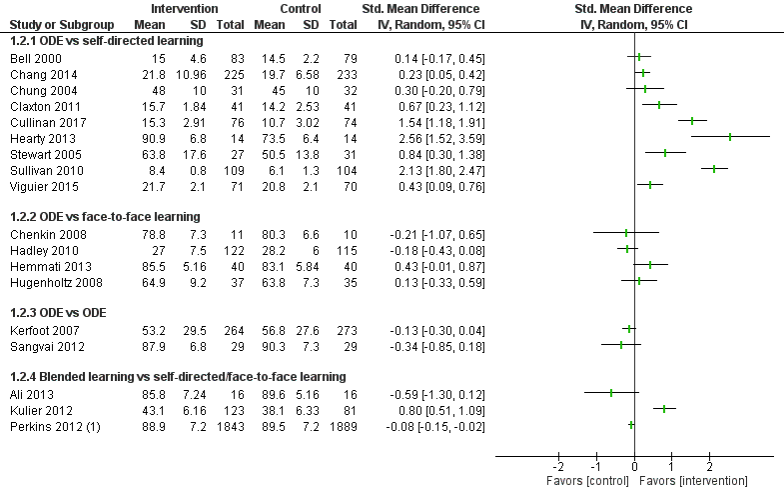
Comparison of postintervention knowledge scores. ODE: online and local area network–based digital education; IV: inverse variance.

A total of 29 studies compared ODE and self-directed learning; of these studies, only 18 studies reported numerical data in a format that could be used (Figures 5 and 6). Eleven studies [43-50,61,63,64] presented incomplete data (missing means, SDs, or CIs), which could not be included in the data analysis. Seventeen studies (n=2107) [43-60] reported that the ODE was significantly more effective than self-directed learning (small to large effect size, very low certainty evidence). Nine studies (n=796) reported that ODE was as effective as self-directed learning (very low certainty evidence). Two studies [61,62] reported mixed results (very low certainty evidence).

##### Online and Local Area Network–Based Digital Education Versus Face-to-Face Learning

Nine studies compared ODE with face-to-face learning; of these, only four studies [83-86] reported numerical data in a useable format (Figure 6). ODE improved physicians’ knowledge compared with face-to-face learning in studies by Fordis et al [81] and Pelayo-Alvarez et al [82]. Six studies [83-88] reported that ODE may be equally effective as face-to-face learning for improving knowledge scores (n=489). McLeod et al [89] reported that knowledge scores were higher with face-to-face learning than with ODE. Overall, empirical evidence suggests that ODE may have little effect on knowledge as compared with face-to-face learning. However, since the evidence was of very low quality, we are uncertain about the true estimate.

##### Blended Learning Versus Self-Directed/Face-to-Face Learning

Seven studies assessed learners’ knowledge, of which two studies [96,97] reported that blended learning was significantly more effective in improving physicians’ knowledge than self-directed/face-to-face learning (n=232; large effect size, very low certainty evidence; Figures 5 and 6). Five studies assessed together [96,98-101] reported that blended learning was as effective as self-directed/face-to-face learning for improving physicians’ knowledge scores (very low certainty evidence).

#### Skills

Twenty-one studies assessed participants’ skills: five studies used an objective structured clinical examination, five studies used questionnaires, four studies used practical skills test/exam, three studies used checklists, and the remaining used other methods to assess skills (Multimedia Appendix 6).

##### Online and Local Area Network–Based Digital Education Versus Self-Directed Learning

**Figure 7:**
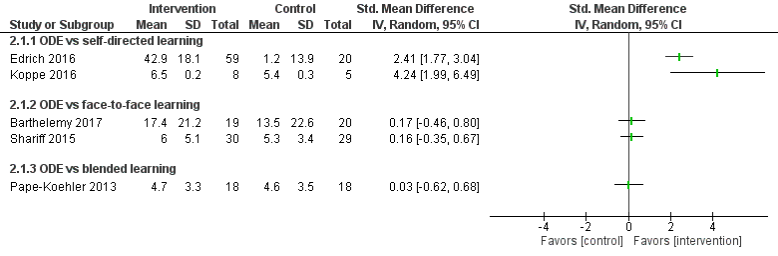
Comparison of change in skills scores (postintervention). ODE: online and local area network–based digital education; IV: inverse variance.

**Figure 8:**
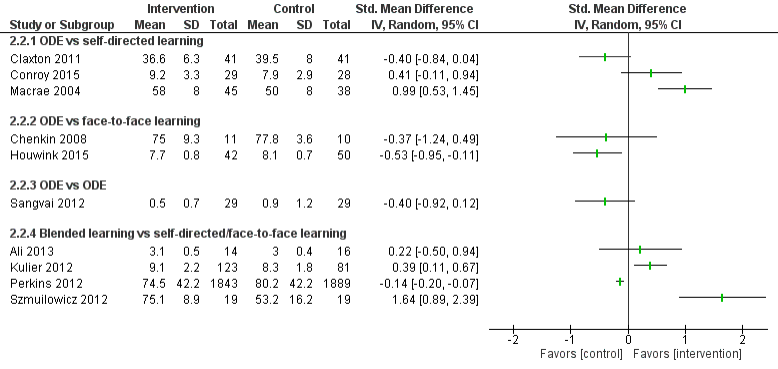
Comparison of postintervention skills scores. ODE: online and local area network–based digital education; IV: inverse variance.

**Figure 9:**
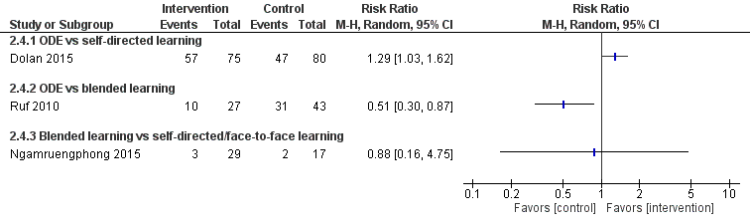
Comparison of postintervention skill scores (dichotomous). ODE: online and local area network–based digital education; M-H: Mantel-Haenszel.

Eight studies [54,65,73-78] compared ODE with self-directed learning, which included no intervention or text-based learning. Of these, only four studies [65,74,76,78] had numerical information in a presentable format (Figures 7-9). Five studies [65,73-76] reported that ODE was significantly more effective than self-directed learning (low certainty evidence). Two studies [77,78] reported that ODE was as effective as self-directed learning (low certainty evidence). One study [54] reported that self-directed learning was more effective than ODE (low certainty evidence). Overall, empirical evidence from five studies [65,73-76] indicated that ODE interventions can improve physicians’ skills as compared to self-directed learning. Similarly, evidence from two [54,77] of the eight studies indicated that ODE may be as effective as self-directed learning for improving physicians’ postintervention skills scores.

##### Online and Local Area Network–Based Digital Education Versus Self-Directed Learning

Seven studies [84,87,90-94] compared ODE and face-to-face learning (classroom didactic lecture–based learning), of which six studies [84,87,90-93] reported that ODE was as effective as face-to-face learning in improving physicians’ skills (low certainty evidence). In one study [94], data were missing (Figures 7-9). Overall, empirical evidence from six studies suggests that ODE may be as effective as face-to-face learning in improving physicians’ skills.

##### Blended Learning Versus Self-Directed/Face-to-Face Learning

Six studies assessed skills [96,98,99,102-104]: Kulier et al [96] and Szmuilowicz et al [102] reported that blended learning may improve physicians’ skills compared with self-directed / face-to-face learning (Figure 8). Overall, empirical evidence from two of the six studies suggests that blended learning may be as effective as self-directed/face-to-face learning in improving physicians’ skills (moderate to large effect size; high certainty evidence). Evidence from four [98,99,103,104] of the six studies suggests that blended learning may be as effective as self-directed/face-to-face in learning to improve physicians’ skills.

#### Attitude

Eight studies assessed participants’ attitude: six studies used questionnaires and two studies [66,105] used Likert scales.

##### Online and Local Area Network–Based Digital Education Versus Self-Directed Learning

Four studies [44,47,58,66] compared ODE with self-directed learning, of which only two studies [58,66] reported numerical data. Le et al [66] reported a change in mean attitude scores (SMD 0.46, 95% CI –0.38 to 1.30 [low certainty]) and Sullivan et al [58] assessed attitude posttest (SMD –0.01, 95% CI –0.28 to 0.26 [low certainty]). Harris et al [47] reported that ODE interventions may improve physicians’ attitude compared to self-directed learning. Le et al [66] reported that ODE may be as effective as self-directed learning for improving physicians’ attitude. Connolly et al [44] and Sullivan et al [58] reported mixed results. Overall, empirical evidence from the four studies reported mixed results for this outcome.

##### Online and Local Area Network–Based Digital Education Versus Face-to-Face Learning

Two studies [82,95] compared ODE with face-to-face learning (classroom didactic lecture–based learning). Only Putnam et al [95] reported a change in learners’ attitude as a dichotomous outcome (RR 0.94, 95% CI 0.72-1.22 [small effect size, low quality]); the other study did not provide data for inclusion in the analysis. Overall, empirical evidence from the two studies reported mixed results for this outcome.

##### Blended Learning Versus Self-Directed/Face-to-Face Learning

Kulier et al [105] compared an integrated ODE course with face-to-face training on evidence-based medicine among obstetrics and gynecology residents; another study by Kulier et al [96] compared an integrated ODE course with a self-directed course on evidence-based medicine and assessed attitude scores at baseline only. Kulier et al [105] reported that blended learning may be as effective as face-to-face training for improving physicians’ attitude (RR 3.00, 95% CI 0.76-11.88).

#### Satisfaction

Sixteen studies assessed participants’ satisfaction, of which 10 studies used questionnaires and 6 studies used Likert scales.

##### Online and Local Area Network–Based Digital Education Versus Self-Directed Learning

**Figure 10:**
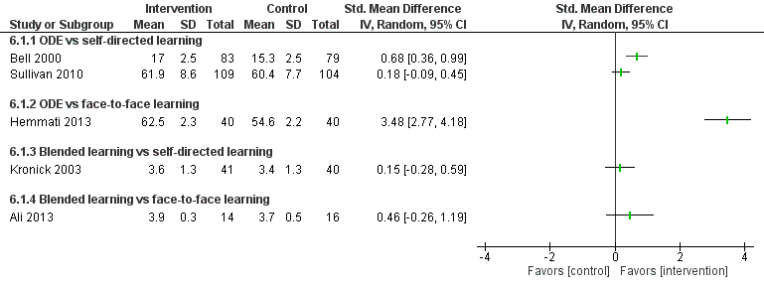
Comparison of postintervention satisfaction scores. ODE: online and local area network–based digital education; IV: inverse variance.

**Figure 11:**
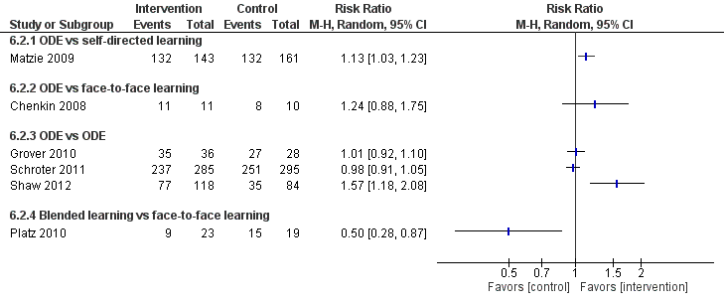
Comparison of postintervention satisfaction scores (dichotomous). ODE: online and local area network–based digital education; M-H, Mantel-Haenszel.

Six studies [54,58,61,67,79,80] compared ODE with self-directed learning, which included no intervention or text-based learning. Of these, only three studies [58,67,79] reported numerical data in a useable format (Figures 10 and 11). Two studies [58,67] assessed mean satisfaction scores posttest (Figure 10). Bell et al [67] reported higher satisfaction for the ODE group than the self-directed learning group (SMD 0.68, 95% CI 0.36-0.99 [moderate effect size, low quality]). Sullivan et al [58] reported no difference in satisfaction scores between the groups (SMD 0.18, 95% CI –0.09 to 0.45). Similarly, Matzie et al [79] assessed posttest satisfaction as a dichotomous outcome (Figure 11) and reported higher satisfaction in four of the five domains (RR 1.13, 95% CI 1.03-1.23 [small effect size, low certainty evidence]). Overall, empirical evidence from two studies [67,79] suggests that ODE may be as effective as self-directed learning on physicians’ satisfaction (moderate to large effect size, low quality). Similarly, evidence from four [54,58,79,80] of the six studies suggests that ODE may be as effective as self-directed learning in improving physicians’ satisfaction. Gold et al [61] reported mixed results.

##### Online and Local Area Network–Based Digital Education Versus Face-to-Face Learning

Four studies [81,83,84,87] compared ODE with face-to-face learning. Of these, two studies [83,84] reported numerical data in a useable format (Figures 10 and 11). Overall, empirical evidence from two [83,87] of the four studies suggested that ODE may be effective compared with face-to-face learning in improving physicians’ satisfaction (large effect size, low certainty evidence). Similarly, evidence from two [81,84] of the four studies suggested that ODE may be as effective as face-to-face learning in improving physicians’ satisfaction.

##### Blended Learning Versus Self-Directed/Face-to-Face Learning

Three studies [98,100,106] assessed satisfaction (Figures 10 and 11). Ali et al [98] reported no difference in satisfaction between the groups, while Kronick et al [106] reported mixed effects. Platz et al [100] found higher satisfaction with face-to-face learning compared to blended learning (moderate effect size, very certainty evidence). Overall, empirical evidence from three [98,100,106] studies suggests that the interventions have mixed effects on learners’ satisfaction.

#### Secondary Outcomes

##### Practice or Behavior Change

Fourteen studies assessed practice or behavior change: four studies used questionnaires; four studies used hospital chart audits or case note reviews; and four other studies used a Likert scale [66], an Intimate Partner Violence Survey scale [64], and patient data from an administrative database and a scenario-based decision-support system [110]. Two studies [111,112] did not state the assessment tools used to measure practice or behavior change.

##### Online and Local Area Network–Based Digital Education Versus Self-Directed Learning

**Figure 12:**
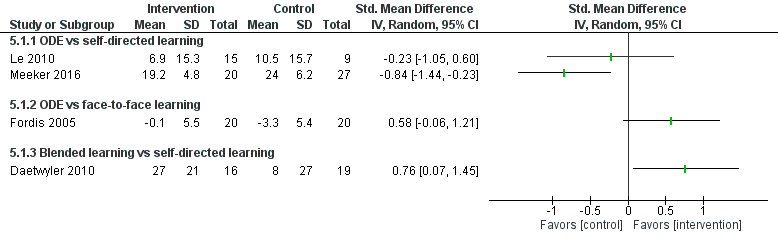
Comparison of practice or behavior change scores (pre-post intervention). ODE: online and local area network–based digital education; IV: inverse variance.

**Figure 13:**
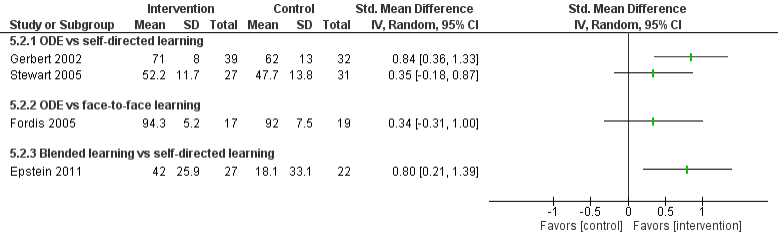
Comparison of postintervention practice or behavior-change scores. ODE: online and local area network–based digital education; IV: inverse variance.

**Figure 14:**
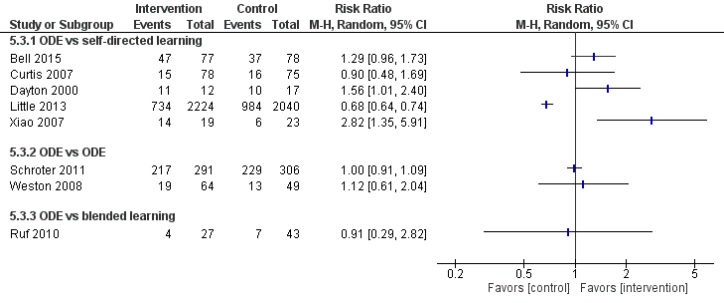
Comparison of postintervention practice or behavior change (dichotomous). ODE: online and local area network–based digital education; M-H: Mantel-Haenszel.

Fourteen studies [43,45,62,64,66,110-118] compared ODE with self-directed learning, which included no intervention or text-based learning. Of these, only nine studies [62,66,110-113,115,117,118] reported numerical data in a useable format (Figures 12-14).

Overall, empirical evidence from 7 [43,45,110,112,114,116,117] of the 14 studies suggests that ODE may be more effective than self-directed learning in improving physicians’ practice or behavior change (moderate to large effect size, very low certainty evidence). Evidence from 4 [62,66,113,115] of the 14 studies suggests that ODE may be as effective as self-directed learning in improving physicians’ practice or behavior change. Three studies [64,111,113] reported mixed results (Figures 12-14).

Fordis et al [81] compared ODE on cholesterol management with face-to-face learning for physicians. The study reported that ODE (online CME) may be as effective as face-to-face learning (live CME) for improving physicians’ practice or behavior change (RR 0.58, 95% CI –0.06 to 1.21; Figures 12 and 13).

##### Blended Learning Versus Self-Directed/Face-to-Face Learning

Of the three studies [104,107,109] that assessed practice or behavior change, only two studies [107,109] reported numerical data; these studies reported mixed results for several behavior change outcomes (Figures 12 and 13). Midmer [104] reported that blended learning may be as effective as face-to-face learning in improving physicians’ practice or behavior change. Overall, empirical evidence from three studies suggests that blended learning may be as effective as self-directed/face-to-face learning.

#### Patient Outcomes for All Comparisons

Four studies assessed patient outcomes: These studies used hospital chart audits [65,114,119] and the European Organization for Research and Treatment of Cancer Quality of Life Questionnaire-C30.

Four studies [65,114,119,120] compared ODE or blended learning with self-directed/face-to-face learning. Butler [114] reported that ODE may be as effective as self-directed learning for improving patient outcomes. Dolan et al [65] reported that ODE may be more effective than self-directed learning for improving patient outcomes (RR 1.07, 95% CI 1.01-1.13). Girgis et al [120] reported that blended learning may be as effective as face-to-face learning for improving patient outcomes (SMD 0.00, 95% CI –0.20 to 0.20). However, Legare et al [119] found that blended learning may be more effective than self-directed learning for improving patient outcomes (RR 2.80, 95% CI 1.44-5.44). Overall, empirical evidence from the two studies [65,114,120] suggests that ODE may be as effective as self-directed/face-to-face learning in improving patient outcomes. In contrast, empirical evidence from two studies [65,119] suggests that ODE/blended learning may be more effective than self-directed learning.

#### Cost

Three studies reported the cost of the ODE interventions [43,99,114]. Braido et al [43] performed inter- or intragroup comparisons and a cost-minimization analysis and reported pharmaceutical cost containment of 29% in the ODE group compared to the self-directed learning group; however, the spending on diagnostic investigations increased by 13.4% in the ODE group and reduced by 24.4% in the control group. Butler et al [114] presented cost information for the Stemming the Tide of Antibiotic Resistance educational program and reported greater reduction in costs postintervention in the ODE group compared to self-directed learning (intervention: £120.76; control: £2.21 per 1000 patients). Perkins et al [99] presented the cost of the Advanced Life Support training program and reported that faculty, catering, and facility costs were 47% lower for the blended learning group than for the conventional training group.

No studies reported on the adverse or unintended effects of ODE or blended interventions.

## Discussion 

### Principal Findings

The review identified a variety of ODE interventions used for postregistration training of medical doctors; these interventions were used among diverse medical specialties, and a range of outcomes and comparators were reported. Because of the high heterogeneity, we were unable to pool the data quantitatively.

A total of 93 RCTs were included, of which 76 studies compared ODE/blended learning with self-directed/face-to-face learning (N=12,424). The results are presented in the main review, and the rest of the comparisons are presented in the Multimedia Appendices 1-13. Among these, 21 studies with 2611 participants reported higher postintervention knowledge scores (small to large effect size, very low quality) for the intervention group, while 20 studies with 5496 participants reported no difference in knowledge scores between the groups. Seven studies with 794 participants reported higher postintervention skill scores in the intervention group (large effect size, low quality), while 13 studies with 4447 participants reported no difference in skill scores between the groups. One study with 99 participants reported higher postintervention attitude scores with the intervention (very low quality), while 4 studies with 305 participants reported no difference in attitude scores between the groups. Four studies with 677 participants reported higher postintervention physician satisfaction with the intervention (large effect size, low quality), while 6 studies with 478 participants reported no difference in satisfaction between the groups. Eight studies with 5051 participants reported higher postintervention practice or behavior change for the ODE group (small to moderate effect size, low quality), while 5 studies with 377 participants reported no difference in practice or behavior change between the groups. One study with 449 participants reported higher improvement in patient outcome, while 3 studies with 667 participants reported no difference in patient outcome for the groups. None of the included studies reported any unintended/adverse effects of the interventions on learners.

Overall, the effect of ODE on postintervention knowledge, skills, attitude, satisfaction, practice or behavior change, and patient outcomes was inconsistent and ranged mostly from no difference between the intervention groups to higher postintervention score in the ODE/blended learning group (small to large effect size, very low to low quality evidence). Moreover, the quality of evidence according to GRADE criteria was judged to be low for most outcomes.

George et al assessed the effectiveness of ODE for undergraduate health professionals [10] and reported that ODE is equivalent, and possibly superior, to traditional learning. We are not aware of any other systematic reviews of RCTs that have evaluated ODE for medical doctors. A similar review of evidence from nonrandomized studies on the effectiveness of ODE in surgical education among medical and dental students, surgeons, and oral health specialists [121] reported knowledge gain from ODE compared with active control (face-to-face learning) or no intervention (self-directed learning). Another review [122] evaluated the evidence of effectiveness from nonrandomized studies of online CME for general practitioners alone; assessed their satisfaction, knowledge, clinical practice, and patient outcomes; and reported improvement in satisfaction, knowledge, or practices. Jwayyed et al [123] evaluated the effectiveness of ODE from nonrandomized studies among diverse health care professionals and reported inconsistent results. Our review, in congruence with other reviews [121-124], compared the effect of ODE and blended learning with self-directed/face-to-face learning and other forms of ODEs on physicians’ knowledge, skills, attitudes, satisfaction and clinical practice, and patient outcomes, but only included evidence from RCTs and cRCTs.

According to the GRADE criteria, the quality of the evidence was very low for knowledge and low for the other primary and secondary outcomes due to the unclear and high risk of bias, inconsistency, and publication bias. The majority of the studies did not provide information on randomization sequence generation and allocation concealment. Similarly, a high proportion of studies (75 studies, 82%; including comparisons presented in the Multimedia Appendix 12) did not provide sufficient information on the blinding of outcome assessors and were hence judged to have an unclear risk of bias. Thirty-one studies (33.3%) reported incomplete outcome data, 25 studies (26.9%) reported baseline differences in participant characteristics and were judged to be at high risk of bias, and 3 studies (3.3%) had a high risk of reporting bias.

This review has a few limitations. The included studies were heterogeneous in terms of the participants, learning content, and the types of ODE (CME/CPD), thus limiting the opportunity to pool the results and consequently run the preplanned subgroup analysis. Hence, the review could not generate conclusive findings on the effectiveness of ODE. Of the included studies, only two were from low- to middle-income countries, which could limit the completeness of the evidence and its generalizability in all settings. Third, we were unable to assess the cost-effectiveness of ODE as compared to self-directed/face-to-face learning, because none of the identified studies formally assessed it. Only three studies assessed the cost and maintenance of the ODE intervention. Fourth, none of the studies specifically addressed any adverse effects of ODE.

The study has several strengths including a thorough and reproducible search of available literature, independent screening and data extractions, and critical appraisal of the literature conducted in accordance with the Cochrane Handbook for Systematic Reviews of Interventions.

### Implications

Given the strength of the evidence of ODE and blended learning, especially for cognitive, procedural, and diagnostic training; evidence-based medicine training; and CME, CPD, and Clinical Practice Guideline training, further high-quality studies of ODE interventions with a validated learning theory are needed to establish their effectiveness, cost-effectiveness, and financial sustainability (return on investment). Specifically, these studies should seek to address several unanswered questions such as ODE’s theoretical underpinning, content validation, frequency, intensity, interactivity, technical features, fidelity, safety, cost-effectiveness, adaptability, acceptability, barriers/facilitators to its adoption, financial sustainability, and learner’s readiness to switch from classroom learning to complete ODE.

### Conclusions

Our review found that ODE and blended learning refers to a group of heterogeneous interventions with different learning theories, learning content, comparators, and outcomes. These interventions were used to train medical doctors in various specialties, such as primary care practitioners, surgeons, residents, and physicians. Although empirical evidence from a majority of studies shows that ODE and blended learning may improve practicing physicians’ knowledge, skills, attitude, satisfaction, practice or behavior change, and patient outcomes, few other studies showed that they may be comparable to self-directed, face-to-face learning. The quality of the evidence in these studies was found to be low and very low for knowledge. Therefore, further high-quality RCTs are required before the evidence of efficacy can be concluded for postregistration training of medical doctors.

## Appendix

Multimedia Appendix 1 Medline (Ovid) search strategy.

Multimedia Appendix 2 Inclusion and exclusion criteria.

Multimedia Appendix 3 Effects of the intervention (other comparisons).

Multimedia Appendix 4 Risk of bias for cluster randomized controlled trials.

Multimedia Appendix 5 Characteristics of included studies assessing knowledge.

Multimedia Appendix 6 Characteristics of included studies assessing skills.

Multimedia Appendix 7 Characteristics of included studies assessing attitude.

Multimedia Appendix 8 Characteristics of included studies assessing satisfaction.

Multimedia Appendix 9 Characteristics of included studies assessing practice or behavior change.

Multimedia Appendix 10 Characteristics of included studies assessing patient outcomes.

Multimedia Appendix 11 Online and local area network–based digital education intervention type by participants’ specialty, country of publication, and intervention duration.

Multimedia Appendix 12 Risk of bias.

Multimedia Appendix 13 Acronyms and definitions.

Multimedia Appendix 14 Effects of the intervention (other comparisons).

## Abbreviations

ATLS: Advanced Trauma Life Support
CME: continuing medical education
CPD: continuing professional development
cRCT: cluster-randomized controlled trial
EBM: evidence-based medicine
GRADE: Grading of Recommendations, Assessment, Development and Evaluations
ICT: information communication technology
LAN: local area network
ODE: online and local area network–based digital education
OSCE: objective structured clinical examination
RCT: randomized controlled trial
RR: risk ratio
SMD: standardized mean difference
