# Supplementary material for: Online Digital Education for Postregistration Training of Medical Doctors: Systematic Review by the Digital Health Education Collaboration
Source: J Med Internet Res. 2019 Feb 25;21(2):e13269. doi: 10.2196/13269 (PMC6410118; doi:10.2196/13269)
Supplement: Multimedia Appendix 1 [file jmir_v21i2e13269_app1.pdf]

Multimedia Appendix 1: Medline (Ovid) search strategy

1. exp education, professional or not education, veterinary or
2. Education, Predental or
3. Education, Premedical or
4. exp Students, Health Occupations or
5. ((medic* or premedic* or dent* or laborat or* or predent* or midwi?e* or nurs* or nutrition* or orthop* or podiat* or pharmac* or psycholog* or psychiatr* or health or healthcare or occupational therap* or physiotherap* or physical therap* or clinical or surg* or radiolog* or obstetric* or gyn?ecolog* or orthodont* or An?esthesi* or Dermatolog* or Oncolog* or Rheumatolog* or Neurolog* or Patholog* or P?ediatric* or Cardiolog* or Urolog*) adj3 (student* or graduate* or undergraduate* or staff or personnel or practitioner* or clerk* or fellow* or internship* or residen* or educat* or train* or novice* or tutor*)).tw,kf.
6. oror1-5
7. Computer-Assisted Instruction or
8. exp Internet or
9. Computer Simulation or
10. Patient Simulation or
11. software or
12. Mobile Applications or
13. User-Computer Interface or
14. Video Games or
15. Web Browser or
16. Education, Distance or
17. Computers or
18. exp Microcomputers or
19. exp Cell Phones or
20. Games, Experimental or
21. exp Models, Anatomic or
22. Audiovisual Aids or
23. Educational Technology or
24. Electronic Mail or
25. exp Telemedicine or
26. Telenursing or
27. Telecommunications or
28. Webcasts or
29. exp Videoconferencing or
30. ((computer* or digital* or hybrid or blended or mixed mode or distance or remote* or electronic or mobile or online* or interactiv* or multimedia or internet or web* or virtual* or game* or gaming or Videogame* or Videogaming) adj3 (classroom* or course* or educat* or instruct* or learn* or lecture* or simulat* or train* or teach* or tutor* or platform*)).tw,kf.
31. (Simulat* adj3 (course* or educat* or instruct* or learn* or train* or teach* or platform* or high-fidelity)).tw,kf.
32. e-learn*.tw,kf.

33. elearn*.tw,kf.
34. m-learn*.tw,kf.
35. mlearn*.tw,kf.
36. smartphone*.tw,kf.
37. smart-phone*.tw,kf.
38. ((mobile or cell) adj2 phone*).tw,kf.
39. iphone*.tw,kf.
40. android*.tw,kf.
41. ipad*.tw,kf.
42. Personal digital assistant*.tw,kf.
43. handheld computer*.tw,kf.
44. Mobile App?.tw,kf.
45. Mobile Application?.tw,kf.
46. webcast*.tw,kf.
47. webinar*.tw,kf.
48. flipped classroom*.tw,kf.
49. Serious game*.tw,kf.
50. Serious gaming.tw,kf.
51. Patient Simulat*.tw,kf.
52. Virtual patient*.tw,kf.
53. ((educat* or instruct* or learn* or simulat* or train* or teach* or interactiv*) adj2 technolog*).tw,kf.
54. Massive Open Online Course?.tw,kf.
55. Mooc?.tw,kf.
56. (Canvas network or Coursera or Coursesites or edx or Futurelearn or iversity or miriada x or moodle or novoed or openlearning or open2study or plato or spoc or udacity or pingpong).tw,kf.
57. oror7-56
58. 6 and 57
59. Education.fs.
60. Education or
61. Teaching or
62. Learning or
63. exp Inservice Trainingor
64. Curriculum or
65. educat*.tw,kf.
66. learn*.tw,kf.
67. train*.tw,kf.
68. instruct*.tw,kf.
69. teach*.tw,kf.
70. oror59-69
71. Health Personnel or
72. exp Allied Health Personnel or
73. Anatomistsor
74. "Coroners and Medical Examiners"or
75. exp Dental Staff or

76. exp Dentists or
77. Health Educators or
78. Infection Control Practitioners or
79. Medical Laboratory Personnel or
80. exp Medical Staff or
81. exp Nursesor
82. exp Nursing Staff or
83. Personnel, Hospital or
84. Pharmacists or
85. exp Physicians or
86. Physician*.tw,kf.
87. Doctor*.tw,kf.
88. Nurs*.tw,kf.
89. Surg*.tw,kf.
90. Health Personnel.tw,kf.
91. healthcare professional*.tw,kf.
92. radiolog*.tw,kf.
93. dentist*.tw,kf.
94. Pharmacist*.tw,kf.
95. Hospital Administrator*.tw,kf.
96. Podiatr*.tw,kf.
97. Psycholog*.tw,kf.
98. Psychiatr*.tw,kf.
99. An?esthesi*.tw,kf.
100. Clinician*.tw,kf.
101. Dermatolog*.tw,kf.
102. General practioner*.tw,kf.
103. Cardiolog*.tw,kf.
104. Oncolog*.tw,kf.
105. Rheumatolog*.tw,kf.
106. Neurolog*.tw,kf.
107. Patholog*.tw,kf.
108. P?ediatric*.tw,kf.
109. Physiotherap*.tw,kf.
110. Physical therap*.tw,kf.
111. Occupational therap*.tw,kf.
112. dieti?ian*.tw,kf.
113. Dietetic*.tw,kf.
114. midwi?e*.tw,kf.
115. nutrition*.tw,kf.
116. orthopti*.tw,kf.
117. obstetric*.tw,kf.
118. gyn?ecolog*.tw,kf.
119. orthodont*.tw,kf.
120. Urolog*.tw,kf.
121. oror71-120

122. Health Occupations or
123. exp Allied Health Occupations or
124. Biomedical Engineering or
125. Chiropractic or
126. exp Dentistry or
127. exp Evidence-Based Practice or
128. exp Medicine or
129. exp Nursing or
130. Dietetics or
131. Optometry or
132. Orthoptics or
133. exp Pharmacologyor
134. exp Pharmacy or
135. Podiatry or
136. Psychology, Medical or
137. Serology or
138. Specialization or
139. exp Surgical Procedures, Operative or
140. exp Radiography or
141. oror122-140
142. 121 or 141
143. 57 and 70 and 142
144. Psychomotor Performance or
145. motor skills or
146. ((psychomotor or procedural or technical) adj3 skill*).tw,kf.
147. (psychomotor adj3 performance).tw,kf.
148. or or144-147
149. 6 and 148
150. 58 or 143 or 149
151. limit 150 to yr="1990 -Current"
