# Supplementary material for: Online Digital Education for Postregistration Training of Medical Doctors: Systematic Review by the Digital Health Education Collaboration
Source: J Med Internet Res. 2019 Feb 25;21(2):e13269. doi: 10.2196/13269 (PMC6410118; doi:10.2196/13269)
Supplement: Multimedia Appendix 2 [file jmir_v21i2e13269_app2.pdf]

Multimedia Appendix 2: Inclusion and Exclusion Criteria

We included studies that evaluated:

- Digital educational interventions (computer-based, computer-assisted) where the learning content was delivered using the Internet or LAN[102] with or without a learning management system (LMS);
- interventions such as web-based tutorials (the online equivalent of classroom-based lectures), discussion boards, email, and Internet-mediated videoconferencing [102];
- educational interventions that required Internet connectivity throughout the duration of the intervention and CMEs that required occasional Internet connections for online discussion and evaluation; and
- educational interventions targeted at 'physician activation'.

We included studies that made the following intervention comparisons:

- online and LAN-based ODE interventions versus traditional learning; and
- blended interventions where online and LAN-based ODE was used together with other forms of intervention versus traditional learning.

The following studies were excluded:

- Studies of digital education that assessed motor skills and skills-based learning (psychomotor domain) were excluded, as they will be analyzed in a separate review.
- studies with mixed participant groups (doctors, nurses, pharmacists), as well as pre- and post-registration healthcare professionals, in which results were not presented separately for each professional group;
- studies of educational interventions targeted at 'patient activation' alone;
- studies of interventions that only required an Internet connection for the downloading of software, slides or other educational content;
- studies investigating telemedicine, telehealth-based learning interventions and video conferencing, delivered through an analogue or digital telephone network or using satellite connectivity;
- studies investigating computer-based educational interventions (e.g., CD-ROMs), unless these interventions used the Internet to disseminate information [102];
- studies in which the digital education was accessed using mobile phones or tablets, as these interventions are covered in a separate review [103];
- studies investigating offline and computer-based digital education among medical students, as these interventions are covered in a separate review [104];
- studies investigating virtual patient simulations in the education of health professionals, as these interventions are covered in a separate review [105];
- studies investigating virtual reality environments in the education of health professionals, as these interventions are covered in a separate review [106];

- studies investigating serious gaming and gamification interventions in the education of health professionals [107]; and
- studies investigating offline and computer-based digital education of medical doctors, as these interventions are covered in a separate review [108].
