# Supplementary material for: Online Digital Education for Postregistration Training of Medical Doctors: Systematic Review by the Digital Health Education Collaboration
Source: J Med Internet Res. 2019 Feb 25;21(2):e13269. doi: 10.2196/13269 (PMC6410118; doi:10.2196/13269)
Supplement: Multimedia Appendix 3 [file jmir_v21i2e13269_app3.pdf]

Multimedia Appendix 3: Effects of Intervention (Other comparisons)

Knowledge

ODE vs ODE

Learners' knowledge was reported in eight studies [110-117] that compared online and LAN-based ODE with other forms of ODE, see Characteristics of included studies assessing knowledge, Appendix VIII. Two studies [112, 114] compared post-test scores between the intervention groups, Figure 2.7. Four studies [110, 113, 115, 117] presented incomplete data (missing means, SDs or confidence intervals), which could not be included in the data analysis. Of the four studies which reported numerical information two studies [111, 116] reported change scores and two [112, 114] reported post-intervention scores and reported that ODE may be equally as effective as the other evaluated ODE for improving physicians' knowledge scores, figures 7 and 8.

Overall, six studies reported than ODE may be equally as effective as the other evaluated ODE for improving physicians' knowledge scores. Three of these studies [112, 114, 117] had a high risk of bias in one of the risk of bias domains.

ODE vs blended learning

Of the three studies that compared blended learning to ODE [118-120] only Talib *et al.* [120] reported a change in knowledge scores for the whole group and hence could not be included in the data analysis. The study reported that ODE intervention may improve physicians' knowledge compared with blended learning (see Characteristics of included studies assessing knowledge, Appendix VII).

Skills

ODE vs other types of ODE

Bernstein *et al.* [110] and Sangvai *et al.* [114] assessed skills among paediatric residents. The intervention in the studies included an interactive web-based module vs a non-interactive module on clinical practice in injury prevention for paediatric residents [114]; and the Bright Futures Oral Health online curriculum (vs ODE on a different topic) [110]. Both studies reported that ODE intervention may improve physicians' skills compared to the other ODE intervention evaluated. Only Sangvai *et al.* [114] reported numerical data format for analysis, Random SMD: -0.40, 95% CI: -0.92-0.12.

ODE vs blended learning

Three studies assessed skills [118-120], of these only two studies [118, 119] reported numerical data in a useable format, Pape-Koehler *et al.* [118] reported that ODE may be as effective as blended learning (combination training) for improving physicians' skills, SMD: 0.03, (95% CI: -0.62-0.68. Ruf *et al.* [119], M-H Random RR: 0.51, 95% CI: 0.30-0.87 and Talib *et al.* [120] reported that blended learning may improve physicians' skills compared to ODE (large effect size; low quality). Overall, empirical evidence from two of the three studies suggests that blended learning may be effective, or as effective, as ODE in improving physicians' skills.

Attitude

ODE vs other types of ODE

Yardley *et al.* [121] assessed attitude among primary care practitioners in a cRCT, see characteristics of included studies assessing attitude, Appendix IX. The study assessed post-test attitude scores, with mixed results observed between the intervention groups.

Satisfaction

ODE vs other types of ODE

Four studies [111, 116, 117, 121] assessed satisfaction, of these studies that assessed satisfaction, only three studies [111, 116, 117] reported numerical data (post-test, dichotomous outcomes) between the intervention groups. Yardley *et al.* [121] presented incomplete data (missing means, SDs or confidence intervals), which could not be used in the data analysis. He reported that ODE (communications group, combined groups) may be effective in improving physicians' satisfaction compared to the other forms of ODE evaluated (CRP and usual care group); however, the study was at high risk of bias for baseline imbalance. The study by Shaw *et al.* [117] reported mixed results for the interventions compared and had a high risk of attrition bias. Two studies [111, 116] reported ODE may be as effective as the other form of ODE in improving physicians' satisfaction; RR: 0.98, 95% CI: 0.91-1.05[116]; RR: 1.01, 95% CI: 0.92-1.10 [111]; the studies had a low or unclear risk of bias, Figure 3 and 4.

Practice or Behavior change

ODE vs other forms of ODE

Four studies [109, 116, 117, 122] assessed practice or behavior change, see characteristics of included studies assessing practice or behavior change, Appendix XI. Of the four

studies that assessed physicians' practice or behavior change, only two [116, 122] reported numerical data as a dichotomous outcome in a format that could be used in the analysis. Two studies [109, 117] presented incomplete data (missing means, SDs or confidence intervals), which could not be included in the data analysis. Overall, empirical evidence from one [109] of the four studies suggests that ODE may be effective compared to other forms of ODE (Internet-based CME) in improving physicians' practice or behavior change. Schroter *et al.* [116] suggested that ODE may be as effective as the other form of ODE evaluated for improving practice or behavior change, RR: 1.00, 95% CI: 0.91- 1.09. Two studies [117, 122] reported mixed results.

Blended learning vs ODE

Ruf *et al.* [119] evaluated an online quality improvement program for alcohol-related disorders for general practitioners, see characteristics of included studies assessing practice or behavior change, Appendix XI. The study assessed practice or behavior change among primary care practitioners and reported mixed results on practice or behavior change (patient documentation), RR: 0.91, 95% CI: 0.29-2.82.

Patient outcomes

Online ODE vs other ODEs

Estrada *et al.* [23] compared an interactive multi-component web-based CME program with a web-based intervention on diabetes practice guidelines for PCPs, and found that both interventions were equally effective in improving patient diabetes care as assessed by haemoglobin A1c, blood pressure and LDL control. Franchi *et al.* [123] compared interactive ODE to improve drug prescribing (module A, B, C, D, E) with another form

of ODE (a refresher course on the basic notions of geriatric pharmacology [module B]), and found that ODE may be equally as effective as the other ODE intervention evaluated for reducing inappropriate medication and drug-drug interactions, RR: 1.14, 95% CI: 0.96- 1.36. Kerfoot *et al.* [124] compared an online space education game with identical online educational content on hypertension management for primary care clinicians, and its effects on patient medication possession ratios. The study found that ODE may be equally as effective as the other ODE for improving patient outcomes, SMD: 0.00, 95% CI: -0.03-0.03.
