# Supplementary material for: Online Digital Education for Postregistration Training of Medical Doctors: Systematic Review by the Digital Health Education Collaboration
Source: J Med Internet Res. 2019 Feb 25;21(2):e13269. doi: 10.2196/13269 (PMC6410118; doi:10.2196/13269)
Supplement: Multimedia Appendix 4 [file jmir_v21i2e13269_app4.pdf]

Multimedia Appendix 4: Risk of bias for cluster randomised controlled trials

Study ID	Recruitment bias	Baseline imbalance	Loss of clusters	Incorrect analysis	Comparability with individual trials
Allison *et al.* 2005	Low	Low	Unclear	High	Unclear
	Recruitment first occurred at the office level (Phase 1) and then at the physician level (Phase 2). In Phase 1, all potentially eligible offices were invited to participate via facsimile. In Phase 2, an active Internet link to the intervention module was delivered by email to physicians recruited in Phase 1 (pg. 286).	Mean screening rates before, during and after the intervention for the comparison offices were 18.9%, 13% and 12.4% respectively, and for the intervention offices were 16.2%, 13.3% and 15.5% respectively (Figure 2, pg. 287).	Loss of clusters not reported in the study	The analysis did not take the clustering effect into account, which would have introduced a unit of analysis error	Comparison between cRCT and RCT could not be made due to heterogeneity of the intervention content
Bernstein *et al.* 2013	Unclear	High	High	High	Unclear
	Recruitment of a subset of all possible residents from each continuity practice was decided by the co-	Baseline characteristics of residents in the intervention and active control groups were comparable by age,	Two sites withdrew after the randomisation phase as a result of site specific	The analysis did not take the clustering effect into account, which would have	Comparison between cRCT and RCT could not be made due to heterogeneity

	investigator to minimise the research burden at each site. Insufficient information was reported to enable judgement.	gender, race, and resident year at the time of intervention (Table 2). Baseline comparisons of clusters are not presented.	inability to accomplish all study elements. (pg. 136, results).	introduced a unit of analysis error.	of the intervention content.
Butler *et al.* 2012	Low	High	Low	High	Unclear
	Randomisation took place once all practices were recruited and all participating clinicians had provided written consent (Randomisation and blinding, pg. 3).	The study compares the summary of demographic features of practitioners in the intervention and control groups but not across clusters (pg. 8).	Two practices, one in each group, withdrew after randomisation but were included in the intention to treat analyses (Results, pg. 4).	The analysis did not take the clustering effect into account, which would have introduced a unit of analysis error.	Comparison between cRCT and RCT could not be made due to heterogeneity of the intervention content.
Epstein *et al.* 2011	Low	Low	Low	High	Unclear
	To create comparable groups of practices across conditions, matched practice pairs were created according to the size of the practice (i.e., number of paediatricians) and	The study clusters are comparable (Table 1, pg. 1202).	No loss to follow-up of clusters (Figure 1, pg. e1204).	The study reported that statistical analysis did not account for potential clustering because of the small number of practices in the	Comparison between cRCT and RCT could not be made, as there were no other studies that compared blended vs self-directed

	the proportion of patients with Medicaid (pg. e1203).			study (pg. e1205).	learning among the paediatricians.
Estrada *et al.* 2011	Low	High	High	Low	Unclear
	A detailed recruitment plan is presented in the protocol of the study and randomisation occurred online immediately after consent.	The study presented characteristics of patients between the groups but not among clusters (pg. 686).	Figure 1 presents the clusters lost to follow-up. Intention-to-treat analysis was conducted. Only 48 and 47 practices were analyzed out of 102 and 103 practices from the intervention and control groups, respectively.	Generalised linear mixed models accounted for clustering of patients (Statistical approach, pg. 684).	Comparison between cRCT and RCT could not be made due to heterogeneity of the intervention content.
Franchi *et al.* 2016	Low	High	Low	Low	Unclear
	Patients were recruited from internal medicine and geriatric wards of the hospital. The wards participating in the pilot phase remained in the same randomisation arm in the full study.	There were no between-arm statistically significant differences pertaining to socio-demographic variables, risk factors and clinical variables, except for the number of diagnoses (P <	No loss to follow-up of clusters, drop out of patients is reported in Figure 1, pg. 56.	The study assessed the primary end-point (differences between intervention and control arms in the prevalence of subjects with at	Comparison between cRCT and RCT could not be made due to heterogeneity of the intervention content.

		0.0001), Cumulative Illness Rating Scale (SIRS) comorbidity index (P < 0.0001) and CIRS severity index (P < 0.0001) both at admission and discharge.		least one potentially inappropriate medication (PIM) at discharge) by means of a logistic regression analysis without covariates in the intention-to-treat population (pg. 55).	
Feng *et al.* 2013	Unclear	High	High	Low	Unclear
	Information about participant recruitment is inadequately reported.	Baseline comparison between the groups and clusters not reported.	Loss to follow-up of patients reported in Figure 1, pg. 318; however, loss to follow-up of participants by cluster not reported.	The study used a multilevel modelling approach to simultaneously estimate the intervention effects while controlling for the hierarchical nature of data explainable within-clinic correlations and design effects	Comparison between cRCT and RCT could not be made due to heterogeneity of the intervention content.

				resulting from the stratified cluster-randomised design, (pg. 317).	
Hadley *et al.* 2010	Low	High	High	Low	Unclear
	All hospitals in the region that were responsible for postgraduate medical education were recruited for the trial (pg. 289). Randomisation was performed after recruitment and consent was obtained.	Baseline comparisons between the groups and clusters were not conducted (pg. 292).	After randomisation, 36 participants in the classroom group and 31 participants in the ODE groups did not complete baseline MCQs, and 7 learners in the classroom and 3 learners in the ODE group did not complete post-test MCQs (Figure 1, pg. 291)	Analyses were done in an intention-to-treat basis. Between arms comparison was undertaken by means of a generalised estimating equations (GEE) model that accounted for within-cluster (i.e. hospital) correlation of the outcome.	The study did not specify the specialty of learners; hence appropriate comparisons could not be made.
Hymowitz *et al.* 2007	Unclear	High	Unclear	Unclear	Unclear
	The study reported residents were nested within residency sites and residencies served	Baseline comparison of characteristics for standard training and special training are	Loss of clusters not reported in the study.	Generalised linear mixed models were used to adjust for several	Comparison between cRCT and RCT could not be made due

	as the unit of randomisation (pg. 5).	presented in Table 2. Statistical comparison was only done within the two groups not between them and not between clusters (pg. 14).		variables but not between clusters (Statistical analysis, pg. 5).	to heterogeneity of the intervention content.
Kulier *et al.* 2009	Unclear	High	Unclear	Low	Unclear
	Sixty-one postgraduate trainees were included in the analysis: 28 in the intervention group and 33 in the control group (Figure 1).	Baseline comparison of demographic characteristics between the intervention groups and clusters are not presented.	Loss of clusters not reported in the study; however 3 and 6 trainees dropped out from the control and intervention groups, respectively (Figure 1, pg. 4).	Generalised estimating equations (GEE) were performed. This allowed extending linear models to take into account the correlation between individuals in the same cluster.	Comparison between cRCT and RCT could not be made, as there were no other studies that compared ODE vs face-to-face learning among obstetricians and gynaecologists.
Kulier *et al.* 2012	Unclear	High	High	Low	Unclear
	Information about learners' recruitment not reported.	The study compared characteristics for the intervention groups: the two groups were similar in age, year of training, attitudes, and	There was loss of clusters attributable to technical difficulties such as interrupted or	Mixed-effects models were used allowing inclusion of all available data, consistent with the intention-	Comparison between cRCT and RCT could not be made, as there were no other studies

		knowledge (Results, pg. 2222, line 16). However, comparisons between clusters were not made.	limited Internet connection, irregular library or computer access, and other issues (pg. 2224, ln 9).	to-treat approach. Such models account for correlation within clusters and within learners (pg. 2221).	that compared blended vs self-directed learning among obstetricians and gynaecologists.
Legare *et al.* 2012	Unclear	High	High	Low	Unclear
	Internet-based software was used to simultaneously randomise all 12 family practice teaching units to either the intervention group (DECISION+2) or control group. However, information regarding randomisation (e.g. whether it preceded recruitment) is not reported.	Characteristics of patients in family practice teaching units before and after the intervention is reported (pg. E729), however statistical comparison was not done for the intervention groups and clusters.	Three of the initial 12 clusters were lost to follow-up (Figure 1, pg. e727).	The study adjusted for potentially confounding variables, baseline scores, and clustering of patients at the level of the teaching unit (pg. e7300).	Comparison between cRCT and RCT could not be made due to heterogeneity of the intervention content.
Little *et al.* 2013	Low	Low	Low	Low	Unclear
	Physicians and patients were unaware of initial group allocation but the	If only 10 patients were recruited from a practice, the network average was used to	18 practices did not recruit patients and were lost to follow-up.	Multilevel logistic regression modelling was used to assess the	Comparison between cRCT and RCT could not be made due

	masking of physicians or patients to the intervention was not possible (pg. 1176).	avoid the imbalance of randomisation by poorly estimated antibiotic prescribing proportions for that practice (pg. 1176).	(Figure on pg. 1177), however an intention-to-treat analysis was done.	main outcome and controlled for baseline antibiotic prescribing rates with allowance for clustering by physicians and practice (pg. 1178).	to heterogeneity of the intervention content.
Marsh-Tootle *et al.* 2011	Low	High	High	Unclear	Unclear
	Providers were sent to the intervention or control websites according to a cluster-randomized schedule that was executed on log-in. Providers who completed the log in screen were randomized and considered enrolled. (Methods, pg. 7161).	Table 2 presents demographic and practice characteristics for those who did and those who did not participate in the study (NP), despite being eligible. However, comparisons by clusters were not presented.	Sixty-five providers were enrolled into the intervention arm and 71 into the control arm. For IPs, responses were available from 61 (93.8%) providers at baseline, 57 (87.7%) after the short delay, and 27 (41.5%) after the long delay. For CPs, responses to	Insufficient statistical information.	Comparison between cRCT and RCT could not be made due to heterogeneity in the intervention content.

			vision questions were available from 42 providers (59.2%) after completing all control modules (pg. 7162).		
McLeod *et al.* 2010	Unclear	Low	High	Low	Unclear
	General surgery training programs in the United States were recruited to participate in the trial. Cluster randomisation was used to allocate the programs to the Internet or moderated journal club format groups (pg. 770).	Baseline characteristics such as number of residents, participation in previous journal clubs, currently teaching appraisal skills, faculty with clinical epidemiology training were reported (Table 2, pg. 772).	In the moderate group, 96% of residents completed at least one package. In the Internet group, only 18% of all residents participated and completed any package (pg. 772). Intention-to-treat was not used for analysis.	Mixed models were used, which allow for the control of putative confounders that might not be addressed in randomisation such as the residency year, age, correlation within the program and training of learners (pg. 771).	Comparison between cRCT and RCT could not be made due to heterogeneity of the intervention content.
Meeker *et al.* 2016	Low	Low	Low	Low	Unclear

	Recruitment of participants was carried out before randomisation of clusters (mentioned in Figure 1, pg. 566).	No observable difference in participant characteristics from the primary care practice.	No loss of clusters.	The study used logistic and non-linear regression methods, which give more conservative estimates of effect sizes (pg. 569).	Comparison of the cRCT with other cRCTs or RCTs was not possible as there were no other studies that compared ODE with similar interventions among primary care practitioners.
Ruf *et al.* 2010	Low	High	Low	Low	Unclear
	112 practices were randomized to the three strategy groups by minimization (pg. 71).	Baseline characteristics of the GPs and patients are reported in the study. There were no statistically significant differences on any variables (pg. 72). However, information on clustering was not reported.	The drop-out rate of practices was significantly higher in the GP+nurse group, but there were no differences concerning sex, age, population of the town or city, patients seen per quarter and Internet know-	The clustered structure of the data was accounted for by an adjustment for the clustering effect in patient-based analyses (pg. 72).	Comparison between cRCT and RCT could not be made, as there were no other studies that compared ODEvs blended learning among primary care practitioners.

			how. Intention-to-treat analysis was used.		
Vollmar *et al.* 2010	Low	Low	High	Low	Unclear
	Cluster randomisation took place at the Quality Circles (QC) level (two arms). Stratified randomisation was performed by a statistician separately for small and large QCs (definition for large QCs: 12 or more participating GPs as reported by the QC moderators) (pg. 4).	The study reported the characteristics of learners and clusters in study arm A, B and the control group (pg. 5). Baseline imbalance of clusters was not reported.	Significant loss to follow-up of clusters (in Figure 1, pg. 3). Intention-to-treat analysis was not performed.	Clustering was taken into account and the authors performed an additional analysis of covariance (ANCOVA) (pg. 4).	Comparison between cRCT and RCT could not be made due to heterogeneity of the intervention content.
Yardley *et al.* 2013	Unclear	High	Unclear	Unclear	Unclear
	GP practices were cluster randomized to the intervention group (pg. 3); however, information on whether recruitment preceded randomisation was not	Baseline comparison was not undertaken.	Loss of clusters not reported in the study.	No details given to enable judgement.	Comparison between cRCT and RCT could not be made due to heterogeneity of the intervention content.

	stated.				

ARIA: Allergic Rhinitis and its Impact on Asthma; ATS: American Thoracic Society; CDC: Centers for Disease Control; CME: continuing medical education; CPD: continuing professional development; N/A: not applicable

Risk of bias in included cRCTs

Evidence for risk of bias in 19 cRCTs is presented in Appendix XIV. Twelve studies were rated as having a high risk of bias for baseline imbalance; eight studies were rated as having a high risk of bias for loss of clusters; and three studies were at high risk of bias for incorrect analyses.

Recruitment bias

Seven studies were rated as having an unclear risk of bias on the recruitment bias criterion as these studies reported insufficient information to permit judgement. Ten studies were rated as having a low risk of bias as no evidence of recruitment bias (recruitment rate differences between the groups) was found in these studies.

Baseline imbalance

Twelve studies were rated as having a high risk of bias for baseline imbalance as baseline differences were compared between intervention groups but not clusters. Five studies were rated as having a low risk of bias as no baseline imbalance between the clusters was found in these studies.

Loss of clusters

Eight studies were rated as having a high risk of bias on the loss of clusters criterion because there was a high drop-out rate among clusters and statistical adjustments to counter for the losses were not reported by the authors. Three studies were rated as having an unclear risk of bias on loss of clusters as they reported insufficient information to permit judgement. Six studies were rated as having a low risk of bias as there was no loss of clusters in these studies.

Incorrect analysis

Three studies had a high risk of bias for incorrect analysis as they did not take clustering into account in the data analysis. Three studies were rated as having an unclear risk of bias as they reported insufficient information on the statistical analyses used. Twelve studies were rated as having a low risk of bias as these studies used appropriate statistical analyses (accounting for intracluster correlation), which took into account the effects of clustering.

Comparability with individual trials

All studies were rated as having an unclear risk of bias as the comparison of cluster with individual trials was not possible due to clinical heterogeneity.
