# Supplementary material for: Online Digital Education for Postregistration Training of Medical Doctors: Systematic Review by the Digital Health Education Collaboration
Source: J Med Internet Res. 2019 Feb 25;21(2):e13269. doi: 10.2196/13269 (PMC6410118; doi:10.2196/13269)
Supplement: Multimedia Appendix 5 [file jmir_v21i2e13269_app5.pdf]

Multimedia Appendix 5: Characteristics of included studies assessing knowledge

Study ID	No. of participants / Specialty	Assessment method	ODE type	Control	Post-intervention knowledge scores
ODE vs self-directed learning					
Alfieri *et al.* 2012	36 / Radiation oncology	Test	eLearning radiation oncology module	Self-directed (text-based learning)	**Intervention** (n=19):<br>Pre-test = 35%<br>Post-test = 52%<br>Mean improvement = 17% (P< .05)<br>**Control** (n=17):<br>Pre-test = 37%<br>Post-test = 42%<br>Mean improvement = 5% (P=not significant)
Bell *et al.* 2000	162 / Primary care practitioners	MCQs	SAGE (Self-study Acceleration with Graphic Evidence) or printed materials on acute myocardial infarction	Self-directed learning (printed self-study materials)	**Immediate post-test scores** were similar in SAGE (n=82) and control groups (n=76) (median score = 15.0 vs 14.5, P> .2, not significant.

					Mean and SD was calculated using median and range.<br>**Follow-up after 4-6 moths**:<br>SAGE group: median = 12, 95% CI: 11 to 13.<br>Control group: median = 11, 95% CI: 10 to 12.<br>Control group: mean = 11.8, (SD = 2.5) and SD were estimated from the values.<br>SAGE group: mean = 12 (SD = 2.5).
Braido *et al.* 2012	60 / Primary care practitioners	Questionnaire	Respiratory allergic diseases: monitoring study of GINA and ARIA guidelines (blended CME)	Self-directed learning (text-based learning)	Knowledge improved significantly after training (P< .001, correct answers to key questions + 13%).<br>Training resulted in pharmaceutical cost containment (trained

					general practitioners + 0.5% vs controls + 18.8 %) and greater attention to diagnosis and monitoring (increase in spirometry + 63.4%, P< 0.01).
Butzlaff *et al.* 2004	72 / Primary care practitioners	MCQs	Clinical guidelines via the Internet or CD-ROM	Self-directed learning (text-based learning)	There was no significant knowledge increase in the intervention group (n=38) (April 2001-13, June 2011-15) (P = .69) compared to the control (n=34) (April 2001-13, June 2011-13).
Cabrera-Muffly *et al.* 2015	37 / Otolaryngology	Exam/test	Informative otolaryngology videos module	Self-directed learning (text-based learning)	Significant improvements in the experimental arm vs the control arm in three of the subspecialty sections (facial plastic surgery, otology, and paediatric otolaryngology); overall mixed results.

					Otolaryngology training examination (OTE), otology intervention: OTE scores among residents (n=18) with access to modules: mean difference = 7 (SD = 20.9).<br><br>Control: OTE scores among residents (n=19) with access to modules: mean difference = 4 (SD = 28.7).<br><br>Mean difference and SD estimated from OTE scores from 2012 and 2013.
Chang *et al.* 2014	458 / Multispeciality	MCQs	Asynchronous eLearning curriculum (Group A, Group B modules)	Self-directed learning	Asynchronous eLearning led to improvement in post-test scores from a mean score of 18.45 (95% CI: 17.92 to 18.98) to 21.30 (95%

					CI: 20.69 to 21.91); a large effect (partial g2 = 0.19).
Chung *et al.* 2004	63 / Emergency medicine	MCQs (34 items)	Online bio-terrorism educational resource	Self-directed learning	No difference between pre- and post-test scores among groups at 1-month and 6-months.<br><br>1 month: web intervention (n=24): 48% +/- 10%; control: (n=29) 45% +/- 10%; mean difference: 3.3%; 95% CI: -8.5% to 2.0%).<br><br>6-months: web intervention (n=22): 51% +/- 8% vs control (n=26): 47% +/- 9%; mean difference: 3.9, 95% CI: -8.8% to 1.2%
Claxton *et al.* 2011	82/ General medicine or internal medicine	MCQs	Fast Facts and Concept e-mails	Self-directed learning	Knowledge post-test scores.

					Intervention (n=41): mean: 15.7 (SD = 2.0).<br><br>Control (n=41): mean: 14.2 (SD = 2.8).<br><br>SD was estimated from the median and range.
Connolly *et al.* 2014	56 / Paediatrics	Questionnaire	'Beyond Milestones' online interactive teaching resource	Self-directed learning	Teaching group scored higher on markers pre- to post-A (mean difference z-score = 0.405, P = .033), and compared to the control group, at post-A (mean difference z-score = 1.078, *P*< .000) and post-B (Mean difference z-score = 0.730, P= .005).<br><br>Teaching group scored higher on observational expertise pre- to post-A (mean difference z-score = 0.521, P=.002) and pre- to post-B (mean

					difference z-score = 0.452, P =.022), and compared to the control group, at post-A (mean difference z-score = 1.243, P<.000) and post-B (Mean difference z-score = 1.075, P<.000).
Cullinan *et al.* 2017	146 / Multispeciality	MCQs and case studies	Standard Computerised Revalidation Instrument for Prescribing and Therapeutics (SCRIPT)	Self-directed learning	4-week MCQ intervention group: 15.36, (SD = 2.91); control group: 10.71 (SD = 3.01), 95% CI of the difference: 3.44 to 5.84, P<.0001.
Dolan *et al.* 2015	41 / General medicine or internal medicine	Questionnaire	Adaptive, longitudinal, online formative self-assessment curriculum delivered via MCQs	Self-directed learning	Residents correctly answered questions on bone mineral testing.<br><br>Intervention (n=21): 15/21, 73%.<br><br>Control (n=20): 13/20, 66%.<br><br>P=.04; effect size =0.65.

Enders *et al.* 2006	265 / Public health	Exam/test	Internet-based introductory bio-statistics course	Cooperative learning	Change in cumulative examination scores between the Cooperative group vs Internet group was estimated by linear model: 4.7 (-5.3, 14.7)
Farah *et al.* 2012	45 / General medicine or internal medicine	MCQs	Information aids, decision aids and the Internet (prostate cancer screening)	Self-directed learning	Intervention (n=22)<br><br>Control (n=20)<br><br>Those who read information aids were more knowledgeable (mean score out of 9: 7.45 vs 5.75, P<.0001.
Ferguson *et al.* 2015	41/ Surgery	MCQs	Online short course on frailty	Self-directed learning	Frailty questions answered correctly.<br><br>Intervention (n=20): 93.7%<br><br>Control (n=21): 75.2%, P<.001
Gold *et al.* 2004	69 / Surgery	MCQs	Internet CD-ROM thoracic surgery eLearning system, a hybrid	Self-directed learning (test-based)	No difference between those residents randomized to receive a CD-ROM set and those randomized to

			surgery curriculum		receive the curriculum outline.
Gyorki *et al.* 2013	97 / Multispeciality	Questionnaire, Likert-type scale	Online spaced education program on the impact of a didactic seminar	Self-directed learning	The residents randomized to the spaced education group (n=28) had a significantly higher post-test score than the control group (n=31): 72% vs 67%, P=.03
Harris *et al.* 2002	99/ Multispeciality	MCQs	Interactive case-based domestic violence education program	Self-directed learning	Domestic violence knowledge at baseline: Intervention = 2.40, control = 2.50<br><br>Post-test mean: Intervention = 3.94, control = 3.53.<br><br>Mean score change: Intervention = 0.18, control = -0.04, P<.001
Hearty *et al.* 2013	28 / Orthopaedic	Questionnaire	eLearning surgical training module on the computer enhanced visual learning platform	Self-directed learning (text-based learning)	Preparedness and surgical decision-making: the correct mean test score (and SD) was 90.9% (SD = 6.8%) for the test group (n=14) and

					73.5% (SD = 6.4%) for the control group (n=14).
Houwink *et al.* 2014	80 / Primary care practitioners	Questionnaire	Genetics eLearning CPD module about oncogenetics	Self-directed learning	Between-group difference was indifferent or in favour of the intervention group, starting from 0.034 (Student's *t*-test, P=.34, non-significant) at T0, and increasing to 0.072 (P=.05) at T1and 0.084 (P=.05) at T2.
Kutob *et al.* 2009	122 / Primary care practitioners	Exam/test	Internet-based approach to teaching cultural competence of type 2 diabetes	Self-directed learning	Intervention (n=58): pretest =183.55 (mean); post-test = 192.09, P=.004 Control (n=64): pre-test = 177.58; post-test = 177.84, P=0.907.
Le *et al.* 2010	24 / Paediatrics	Questionnaire	Web-based multimedia learning modules for physicians' knowledge, attitudes and	Self-directed learning	Intervention group (n=11): 4.75 (SD = 3.28); control group (n=9): 2.88 (SD = 2.03), P=.19.

			treatment on paediatric asthma		
Satterwhite *et al.* 2012	17 / Surgery	MCQs	A webpage on microsurgery, entitled ‘Microsurgery Essentials’ (website)	Self-directed learning	For residents who used the webpage (n=9), there was a 17%-point improvement between their pre- and post-test scores, from 62% to 82% (P= .01).<br><br>For residents who did not have webpage access (n=8), there was essentially no difference between their pre- and post-test scores (76% and 75%, respectively, P= 0.80).
Short *et al.* 2006	81 / Multispeciality	Questionnaire, Likert-type scale	Online intimate partner violence CME program	Self-directed learning	There were significant positive changes on perceived knowledge, P=.000). No numerical data was reported.
Stewart *et al.* 2005	58 / Primary care practitioners	Questionnaire	Case-based online learning (CBOLL) group	Self-directed learning	Knowledge score: Intervention (n=27)

					Before (n=27):<br><br>Prevention topic: 53.8 (12.8)<br><br>Diabetes topic: 66.8 (14.1) 2-months after (n=27): Prevention topic: 63.8 (17.6)<br><br>Diabetes topic: 72.7 (14.1)<br><br>6-months after (n=17): Prevention topic: 65.7 (15.2)<br><br>Diabetes topic: 73.2 (7.7)<br>Control (n=31)<br><br>Before (n=31):<br><br>Prevention topic: 51.9 (9.5) Diabetes topic: 68.6 (10.4) 2-months after (n=31): Prevention topic: 50.5 (13.8),<br><br>Diabetes topic: 67.7 (16.8)

					6-months after (n=24):<br>Prevention topic: 53.3 (10.5)<br>Diabetes topic: 68.6 (11.4)
Sullivan *et al.* 2010	213 / General medicine or internal medicine	Questionnaire	Web-based module, opioid therapy for chronic non-cancer pain.<br>COPE: Collaborative Opioid Prescribing Education.	Self-directed learning (Veteran Affairs Opioid Guidelines)	Knowledge test (range: 0-9).<br>Intervention (COPE course; n=109): mean = 8.4 (SD = 0.8).<br>Control (VA guidelines; n =104): mean = 6.1 (SD =1.3).
Thompson *et al.* 2012	347 / Gastroenterolog y	Questionnaire , Likert-type scale	Web-based tutorial (gastrointestinal endoscopy)	Self-directed learning	Total score, mean (SD).<br>No tutorial:<br>Survey 1 = 56.9 (SD = 15)<br>Survey 2 (n=102) = 56.9 (SD= 14<br>Tutorial:

					Survey 1 = 56.4 (SD = 15)<br><br>Survey 2 = 64.5 (SD = 18).
Viguier *et al.* 2015	141 / Rheumatologists	Exams	Online training on skin tumours	Self-directed learning	Primary evaluation criterion (Score 1: diagnosis of the benign vs premalignant/malignant nature of the lesions).<br><br>The means difference in the number of adequate responses at Test 2 between groups was 2.2 points, favouring the online training group (95% CI: 1.3 to 3.1), P<.0001.
Wang *et al.* 2013	44 / Radiology	Questionnaire, Likert-type scale	Computer-based training procedures	Self-directed learning (hands-on simulation training procedures)	Computer group (n=22): mean (range) = 2.5 (0.0, 5.0), P<.00.<br><br>Hands-on group (n=22): mean (range) = 2.5 (0.0, 6.0), P<.001.

					None of the mean scores were significantly different between groups (P>.05 for all).
Westmoreland *et al.* 2010	96 / Multispeciality	Questionnaire	Web-based module (geriatrics content)	Self-directed learning (text-based learning)	Mean change score = 27.6 (SD = 18.1) for residents who completed web-based instruction and 9.0 (SD = 20.0) for residents who completed the paper-based instruction.<br><br>n=48 per group.
ODE vs face-to-face learning					
Bello *et al.* 2005	56 / Anaesthesiology	MCQs	Online teaching	Face-to-face learning	Knowledge gains in Group 2 were slightly, but not significantly, greater compared to Group 1 (n=2): Median from 12.0 to 29.0; Group 2 (n=28), Median from 13.5 to 30.5 in written test, (P=.228).

Chan *et al.* 1999	23 / Primary care practitioners	MCQs	Problem-based small group learning on depression among elderly (face-to-face learning)	Face-to-face learning (small group discussion)	Mean pre-test MCQ score was 66.2 (SD = 7.3); follow-up test score was 67.2 (SD = 10.5).<br><br>No difference in test scores (pre- and post-test combined) based on group allocation.
Chenkin *et al.* 2008	21/ Emergency medicine	Questionnaire	Web-based ultrasound-guided vascular access training	Face-to-face learning (didactic discussion)	No difference in mean written test scores (absolute difference = -1.4%; 95% CI: -7.8% to 5.0%), between the web group and the didactic group, P=.65.
Fordis *et al.* 2005	103 / Primary care practitioners	MCQ and case vignettes with fixed-choice responses	Intervention 1: Online CME on cholesterol management	Live interactive CME	Online CME group (n=44) scored slightly higher than the live CME group (n=49) when averaged across all three testing occasions (4.8% additional items correct, 95% CI: 0.6%

					to 9.0%; partial ω2= 0.01, P= .03).<br><br>85% of the randomized participants in the online CME group and 96% in the live CME group completed the knowledge tests.
Hadley *et al.* 2010	7 hospitals, 237 trainees/ Not specified	MCQs	A clinically integrated eLearning course for teaching EBM	Face-to-face learning	There was no difference in the improvement of knowledge between the intervention (n=88) (baseline = mean: 22.9, SD: 7.0; post-test mean=27.0, SD: 7.5; and the control (n=72) (baseline= mean: 24.7, SD: 3.9; post-test mean=28.2, SD: 6.0, groups. P=.89
Hemmati *et al.* 2013	80 / Primary care practitioners	Exam/test	CPR curriculum guidelines training through Internet-based learning (Group 2)	Face-to-face learning	No difference in knowledge scores between the intervention (n= 40) (pre-test: 67.87 [SD =5.41]; post-test: 85.5

					[SD = 5.16]), and the control group (n=40) (pre-test: 68.5 [SD = 5.68]; post-test 83.12 [SD = 5.84]).
Hugenholtz *et al.* 2008	72 / Occupational physicians	Questionnaire	Website including an eLearning module on mental health issues	Face-to-face learning	Mean score for the eLearning approach (n=37) was 52.1 (SD = 8.4) at baseline and 65.1 (SD = 9.6) at post-test (P= .05).<br><br>For the lecture-based approach (n=35), the mean score was 52.3 (SD = 9.0) at baseline and 64.3 (SD = 9.0) at post-test, P=.05.
McLeod *et al.* 2010	12 general surgery programs, 441 residents / Surgery	MCQs	Critical appraisal skills (Internet and emails)	Face-to-face learning (didactic discussion)	Completed the appraisal test: Internet group (n=227): 57.8%<br><br>Moderated group (n=216): 72.7 %<br><br>Mean score:

					Moderated group = 43.8% (CI 40.2 to 47.5); Internet group = 39.0 % (CI 35.8 to 42.2); P=0.05, moderate effect size of 0.6 SD.
Pelayo-Alvarez *et al.* 2013	169 / Primary care practitioners	MCQs	Palliative care education (online program)	Face-to-face learning	Knowledge test at 4-months showed a positive difference of 5.2 (95% CI: 3.4 to 6.9) in the intervention group.
ODE vs other types of ODE					
Bernstein *et al.* 2013	29 clusters, 208 paediatric residents/ Paediatrics	MCQs	Bright Futures Oral Health online curriculum	1-hour online curriculum (not related to intended eLearning content)	Intervention group demonstrated significant improvement in general Bright Futures knowledge (Mean-pre =71.4; mean-post=80.4, F=5.296) compared to the control group at 3-months (mean-

					pre=73.9; mean-post=74.3, P=.023).
Grover *et al.* 2010	210 / General medicine or internal medicine	Questionnaire, Likert-type scale	Arterial and central line placement with a web-based curriculum. Intervention 1: Both central venous line and arterial line procedures. Intervention 2: Central venous line but not arterial line. Intervention 3: Arterial line but not central venous line.	Self-directed learning (text-based learning)	Central venous line: Curriculum available (n=28): Pre-test = 64.3%; Test 1 = 65%; Test 2 = 70%; score difference = 5%, effect size/P=.25/.11 Central venous line: No additional curriculum available (n=37): Pre-test = 58.6%; Test 1 = 62.1%; Test 2 = 62.9%; score difference %, 0.8. Arterial line: Curriculum available (n=36): pre-test, 55.5%; Test 1 = 58.4%; Test 2 = 70.3%; score difference = 11.9%, effect size/P=.52, <.001. Arterial line: No additional curriculum

					available (n=49): Pre-test = 58.7%; Test 1 = 63.2%; Test 2 = 63.4%; score difference = 0.2 %; effect size/P value, .52/<.001.
Kerfoot *et al.* 2007	537 / Urology	MCQs	Online educational program on spacing effect principle on the acquisition and retention of medical knowledge (cohort 2)	Bolus cohort	Cohort 1 (n=254), mean (SD):<br><br>Overall percentile ranking = 53.2 (29.5)<br><br>Percentile ranking in 4 topic areas = 54.5(28.2);<br><br>Cohort 2 (n=261), mean (SD):<br><br>Overall percentile ranking = 56.8 (27.6)<br><br>Percentile ranking in 4 topic areas = 57.7(28.2)<br><br>Follow-up at 2 years: Residents in the spaced education cohort had significantly greater

					test scores than residents in the bolus cohort: mean = 70.2% (SD = 9.0%) vs 66.8% (SD = 10.6 %), effect size 0.35, P= .03.
Marsh-Tootle *et al.* 2011	136 providers / Primary care practitioners	MCQs	Strabismus and amblyopia (vision) (website)	Blood Pressure screening and Chlamydia screening	Mean score: Before = 3.1 After (short term; n=57) = 5.5, P<.000 Before = 3.3 After (Long term, n=27) = 3.7, P=0.14; Control (n=42) = 3.0 P=0.49[a]; P<0.000[b]; P=0.03[c] [a] Control vs intervention at baseline [b] Control vs intervention after short term delay

					[c] Control vs intervention after long term delay
Sangvai *et al.* 2012	57 / Paediatrics	MCQs	Motor vehicle safety, bicycle safety, poison prevention, fire/burn prevention, and firearm safety (PowerPoint presentations)	Web-based non-interactive modules	Control group (n = 28) had significantly higher post-test scores (mean = 90.36 [SD = 7.32]) than the intervention group (n = 29) (mean = 87.93 [SD = 6.88]), after adjusting for pre-test scores (control group: mean = 60.0 [SD = 11.06]; intervention group: mean = 64.31 [SD = 8.84]) and year of training (P=.036).
Saxon *et al.* 2015	44 / General medicine or internal medicine	Questionnaire, Likert-type scale	Manuscript with embedded hyperlinks	Manuscript without hyperlinks	Knowledge of selected bio-statistical terms was measured using five questions. Pre- and post-test % of correct responses for Group A and B are reported in the manuscript.

Schroter *et al.* 2011	1054 / Multidisciplinary	Questionnaire	Diabetes Needs Assessment Tool (DNAT)	Diabetes learning modules	The mean knowledge test scores increased similarly in both groups, from 47.4% (SD = 12.6) to 59.0% (SD = 15.8) (n = 499) and 47.3% (SD = 12.9) to 60.1% (SD = 15.9) (n= 498) in the intervention and control groups, respectively. (P= .172).
Shaw *et al.* 2012	371 / Multispeciality	MCQs	Intervention 1: Online spaced education program to improve knowledge and compliance with the National Patient Safety Goal (NPSG). Intervention 2: Slide show based online program to improve knowledge and	Self-directed learning (text-based learning)	Spaced education: mean pre-test score = 12.3; mean post-test score = 13.5; pre-test versus post-test score: P<.000.

			compliance with the NPSG.		
Blended learning vs ODE					
Talib *et al.* 2010	56 / Paediatrics	MCQs	Hands on training + web-based training on oral health counselling	Web-based training on oral health counselling	Compared to the baseline, resident knowledge improved after the web-based training for the whole group from 69% to 81% (Difference = 12% [95% CI: 9% to 15%], P=.001).
Kulier *et al.* 2012	60 training units, 204 post-graduate trainees in obstetrics and gynaecology / Obstetrics and gynaecology	MCQs	A clinically integrated eLearning course incorporating the WHO reproductive health library for teaching basic EBM among postgraduates	Self-directed EBM course	Knowledge (overall MCQ scores): Control intervention: baseline = 38.5 (37.3 to 39.7); post-course = 38.1 (36.7 to 39.4) Experimental intervention: baseline = 38.4 (37.3 to 39.4); post-course = 43.1 (42.0 to 44.1).<br><br>Adjusted mean difference: 4.9 (2.9 to 6.8), P<.001.

Sharma *et al.* 2013	28 / Anaesthesiology	MCQs	Echocardiography learning (website)	Self-directed learning (guidelines)	Post-intervention test scores were higher than pre-test scores in both study groups (Non-Internet group [n=11]: 44 [10]; Internet group [n=12]: 59 [10]), but the increase was greater in the Internet group.<br><br>The increase in the post-simulation test compared to the post-intervention test was significantly greater in the non-Internet group (Non-Internet group [n=11]: 63 [5]; Internet group: [n=12] 72 [8]).
Ali *et al.* 2013	30 / Primary care practitioners (family practice residents)	MCQs	ATLS delivered through telemedicine	ATLS face-to-face learning	Telemedicine (n=14): mean = 85.89 (95% CI: 82.03 to 89.76), P=.091<br>Control (n=16): mean = 89.69 (95% CI: 86.94 to 92.43).

Kulier *et al.* 2009	6 training units 61 post-graduate trainees in obstetrics and gynaecology / Obstetrics and gynaecology	MCQs	A clinically integrated eLearning course for teaching basic EBM among postgraduates	Face-to-face learning	Intervention group outperformed the control group by 3.5 scoring points (95% CI -2.7 to 9.8) but this difference was not statistically significant (P=.27).
Perkins *et al.* 2012	3732 / Multispeciality	Exam/test	Advanced life support (ALS) training (LMS)	Face-to-face learning (conventiona l ALS course)	eALS (n=1403): 74.5%; cALS (n=1445): 80.2% 95% CI: -8.8 to -2.7
Platz *et al.* 2010	55 / Emergency medicine	MCQs	Web-based basic ultra-sonographic principles and the Extended Focused Assessment with Sonography for Trauma (EFAST) (PowerPoint slides)	Face-to-face training	Post-test: Both the classroom (n=19) and Web group (n=23) showed significant improvement in scores between the pre- and post-test 1 (75.9% [SD = 10.7] vs 93.9% [SD = 4.7], with a difference of 18.0% (95% CI 12.5% to 23.5%).
Vollmar *et al.* 2010	389 / Primary care practitioners	Questionnaire	1. Study Arm A (blended learning, online	Face-to-face learning (lecture,	Difference in knowledge gain (t1-t0): Study group A

			modules and structured discussion)<br>2. Study Arm B (lecture, structured discussion)<br>3. Study Arm A and B	face-to -face discussion)	(n=84) and B (n=82) did not show any statistically significant difference in knowledge gain on all 20 questions at T1 (3.67 versus 3.60 questions, mean difference = 0.07, CI: -0.84 to 0.98, P = .881; T=0.15).<br>Difference in knowledge gain (t2-t0): Study group A (n=46) and B (n=51) did not show any statistically significant difference in knowledge gain on all 20 questions at T2 (2.39 versus 2.00 questions, Mean difference = 0.39, CI: -0.83 to 1.61, P = .526; T=0.636).<br>Outcome of control group: The non-randomized control

					group (n=21) also showed an improvement in knowledge, although the knowledge gain at T2 was lower (1.48, P = .019) compared to the intervention groups at both times.

ARIA: Allergic Rhinitis and its Impact on Asthma; ATLS Advanced Trauma Life Support; CME: continuing medical education; CPD: continuing professional development; CPR: cardiopulmonary *resuscitation*; EMB: evidence-based medicine; GINA: Global Initiatives for Asthma guidelines; MCQ: multiple choice question.
