# Supplementary material for: Online Digital Education for Postregistration Training of Medical Doctors: Systematic Review by the Digital Health Education Collaboration
Source: J Med Internet Res. 2019 Feb 25;21(2):e13269. doi: 10.2196/13269 (PMC6410118; doi:10.2196/13269)
Supplement: Multimedia Appendix 6 [file jmir_v21i2e13269_app6.pdf]

Multimedia Appendix 6: Characteristics of included studies assessing skills

Study ID	No. of participants / Specialty	Assessment method	eLearning type	Control	Post-intervention skills
ODE vs self-directed learning					
Claxton *et al.* 2011	82 General medicine or internal medicine	Likert scale	Fast Facts and Concept (FFAC) emails	Self-directed learning	Preparedness in symptom management skills improved more in the intervention group (n=41) than in the control group (n=41) (P= .04, .01, and .02, respectively).<br>Post-test: overall preparedness was 39.5 in the control group (median), and 36 in the intervention group, P=.06.
Conroy *et al.* 2015	57 / Paediatric trainees	Liverpool Causality Assessment Tool	Liverpool ADR Causality Assessment eLearning Package (LACAeP)	Self-directed learning	Intervention: n=29<br>Control: n=28<br>The average score by correct classification was 9.22 (95% CI: 7.96 to 10.48) in the intervention arm and 7.88 in the control arm (95% CI: 6.76 to 9.00).
Dolan *et al.* 2015	41 / General medicine or internal medicine	Survey	Online curriculum in bone health	Self-directed learning (text-based training)	Treatment rates for high risk fragility fractures:<br>Intervention: 57/75, 76%<br>Control: 47/80, 59%<br>P= .03
Edrich *et al.* 2016	138 / Anaesthesiology	Objective structured clinical	1.Web-based training of lung ultrasound for	1. Self-directed learning (text-based learning)	Mean combined test scores:<br>Intervention (Web group; n=59): mean = 42.9 (SD = 18.1)

		examination (OSCE)	the exclusion of pneumothorax	2. Classroom-based training (face-to-face learning)	Classroom group (n=59): mean = 39.2 (SD = 19.2).<br>Intervention (Web group; n=59): mean = 42.9 (SD: 18.1)<br>Control (n=20): mean = 1.2 (SD = 13.9).
Hymowitz *et al.* 2007	14 paediatric residency training sites, 88 residents / Paediatrics	Questionnaire	Seminar on' solutions for smoking' website served as the main teaching tool	Self-directed learning	Intervention: n=44<br>Control: n=44<br>There were no significant differences between the intervention and control groups on the post-test survey results on any of the domains tested.
Koppe *et al.* 2016	14 GPs, 12 GP registrars / Primary care practitioners	Objective structured assessment of technical skills (OSATS)	Balint groups were delivered over 8-9 fortnightly online sessions via WebEx	Self-directed learning	Task-specific checklist:<br>Pre-test:<br>Multimedia-based training (n=18): 6.6 (SD = 2.8)<br>Practical training (n = 17): 5.5 (SD = 3.7)<br>Combined training (n = 18): 5.8 (SD = 3.3)<br>Control group (n = 17): 5.5 (SD = 2.8).
Lee *et al.* 2015	125 / Primary care practitioners	Diagnostic assessment	Online cultural competency and problem-affect-concern-treatment (PACT) training	Self-directed learning (text-based training)	GP group (n=43): Correct diagnosis % = 72.2%<br>GP and Nursing group (n=42): Correct diagnosis % = 69.7%<br>Control group (n=27): Correct diagnosis % = 36.4%

Macrae *et al.* 2004	81 / Surgery	Videotape of encounters	General surgery (Internet, emails)	Self-directed learning (clinical articles)	No difference between pre- and post-intervention. Videotaped encounter scores (P=.432). Both the interactive and non-interactive groups had lower post-intervention than pre-intervention scores. No difference in post-intervention Videotaped encounter scores by group (P=.822).
ODE vs face-to-face learning					
Bello *et al.* 2005	56 / Anaesthesiology	Practical skills test (on manikin)	Online teaching	Face-to-face learning	Skills gains on practical skills tests were slightly, but not significantly, greater in Group 2 (n=28) (31.5% to 46.0) compared to Group 1 (n=28; 32.5% to 47.0%, P=.376.
Barthelemy *et al.* 2017	39 / Emergency medicine	Questionnaire	Online Modular Object-Oriented Dynamic Learning Environment (Moodle) for electrocardiography interpretation	Face-to-Face (lectures)	Skills: ODE group (n=19): precourse = 42.1% (34.8-49.4), (SD = 15.15). Lecture-based group (n=20): precourse = 37.5% (30.7-44.2), P=0.42. ODE group (n=19): postcourse: 59.5% (51.8-67.1), (SD = 15.87); Lecture-based group (n=20): postcourse = 51%, (42.4-59.6), (SD =18.38) P=0.14
Chenkin *et al.* 2008	21 / Emergency medicine	OSCE	Web-based ultrasound-	Face-to-face learning	No difference in mean OSCE scores (absolute difference = -2.8%, 95% CI: = -

			guided vascular access training	(didactic group)	9.3% to 3.8%) between the web group (n=11) and the didactic group (n=10), P=.39.
Houwink *et al.* 2015	92 / Primary care practitioners	Exam/ test	1. Genetics eLearning CPD module about oncogenetics 2. GP and genetics website	Face-to-face (live genetic CPD module) learning	26 (58 %) in the intervention group (n=45) and 29 (76 %) in the control group (n=38); P=0.10. Intervention group mean score = 58% (SD= 8); control group = 50% (SD = 8); *t*=3.92, P <.0001.
Shariff *et al.* 2015	5959 / Surgery	Questionnaire	Multimedia educational tool for cognitive surgical skill acquisition in open and laparoscopic colorectal surgery	Face-to-face (study day group) lectures	Residents in the intervention group were significantly more likely to treat patients at high risk for fragility fracture with bisphosphonates, treating 57 of 75 compared to control group residents who treated 47 of 80 patients (76% versus 59%, P=.03).
Schmitz *et al.* 2016	72/ Surgery	OSCE	Mastering Difficult Family Conversations in Surgical Care online course	Face-to-face learning	Residents in the treatment group improved their OSCE scores from 58.8 to 72.5 (change score = 13.7); residents in the control group improved their OSCE scores from 60.9 to 68.0 (change score =7.0), P>.05.

Wilkinson *et al.* 2016	24 / General medicine or internal medicine	Exam/ test	Technology-driven simulation-based cardiac ultrasonography teaching	Conventional teaching	Post-test scores improved significantly in both groups (P<0.01). The change in scores (mean, [SD]) in the multimedia group (n=30) was not significantly different from the study day group (n=29) (6.02 [SD = 5.12] and 5.31 [SD=3.42], respectively; P = .61).
ODE vs other types of ODE					
Bernstein *et al.* 2013	27 continuity research network sites, 143 paediatric residents / Paediatrics	Structured clinical observations	Bright Futures Oral Health online curriculum	1-hour online curriculum	Intervention: n=52<br>Control: n=60<br>At 3-months, the intervention group demonstrated significant improvement in oral health performance (mean-pre=0.9, mean-post=1.2, *F*=15.220, P<.001), compared to the control group (mean-pre=0.9, mean-post=0.9, P=.001)
Sangvai *et al.* 2012	57 / Paediatrics	MCQs	Motor vehicle safety, bicycle safety, poison prevention, fire/burn prevention, and firearm safety (PowerPoint presentations)	Web-based non-interactive modules	Web-based interactive modules (n=29): Mean = 0.5 (SD= 0.7), Control (non-interactive web-based modules; n=29): Mean = 0.9 (SD = 1.2).

Wilkinson *et al.* 2016	24 / General medicine or internal medicine	Objective structured clinical examination	Motor vehicle safety, bicycle safety, poison prevention, fire/burn prevention, and firearm safety (PowerPoint presentations)	Web-based non-interactive modules	Interpretation ability:<br>Before teaching, both groups could correctly identify a case as grossly normal or abnormal 29% and 27% of the time, respectively. After teaching, this increased to 55% and 65%, respectively (P=.194).<br>Before teaching, both groups were rarely able to make a correct singular diagnosis: 12% in the conventional group and 15% in the technology group.<br>After teaching, the ability to make a singular correct diagnosis increased to 32% and 39% (P=.439) in the conventional and technology groups, respectively.<br>Scanning ability:<br>In the conventional group, 7 of 13 (53.8%) had diagnostic quality images (P=0.006), compared to 2 of 11 (13.6%) in the technology group.
Blended learning vs ODE					
Talib *et al.* 2010	56 / Paediatrics	MCQs	Hands-on-training (HOT) and web-based training (WBT) on oral	WBT on oral health counselling	ODE: n=27<br>Blended learning: n=29<br>WBT+HOT group scored higher than those in the WBT group (87% vs 73%; difference = 13.9%, 95% CI: 1.2% to 26.6%, P=.03)

			health counselling		
Blended learning vs face-to-face learning					
Szmuilowicz *et al.* 2012	38 / General medicine or internal medicine	Behavioral checklist	Internet-based multimodality communication skills intervention	Face-to-face learning (clinical rotation) / self-directed learning	Intervention group (n=19) residents displayed significantly higher overall performance with less variation than control group residents (n=19) on the CSD checklist outcome evaluation (75.1% –8.9 versus 53.2% – 16.2, P <.001).
Midmer *et al.* 2006	88 / Primary care practitioners	Checklist	Opioid- and Benzodiazepine-Prescribing Skills (emails) and discussions	Face-to-face learning (3-hour interactive presentation)	Code status discussions skills total score: Intervention (n=19): (mean = 75.1% [SD = 8.9%])<br>Controls (n=19): (mean = 53.2% [SD = 16.2%] P<.001
Ngamruengphong *et al.* 2015	39 / Primary care practitioners	Checklist	Didactic lecture and periodic email remainders with immediate feedback on	Face-to-face learning (standard education from the residency program)	Skills improvement: Intervention (WBT+HOT group) = 87% vs control (WBT group) = 73%; difference = 13.9%, 95% CI: 1.2% to 26.6%, P=.03)

			HBV vaccination		
Perkins *et al.* 2012	3732 / Multispeciality	Psychological Medicine Inventory, Professional Isolation Scale, Warr's Work-Related Affect Scale	Advanced life support (ALS) training (LMS)	Face-to-face (ALS course)	Balint participants' scores (n=8) were significantly higher post-intervention on the Psychological Medicine Inventory (mean = 6.49 [SD =0.20]) versus the control (n=5) (mean = 5.43 [SD = 0.26], P< .01) and similarly on Warr's Work-Related Affect scale scores: intervention (n=8): mean = 4.09 (SD = 0.09) versus the control (n=5): mean = 3.60 (SD = 0.12), P < .01). Effect size on these scales ranged from 0.46 to 0.50.
Ali *et al.* 2013	30 / Surgery	Instructor	ATLS delivered through telemedicine	Standard ATLS course	Control n=16: 3.125 (SD=0.5)<br>Intervention n=14: 3.00 (SD=0.39), P=.45
Pape-Koehler *et al.* 2013	70 / Surgery	Documentation skills, chart audit	Surgical performance in completing a laparoscopic cholecystectomy in a Pelvi-Trainer (web-site)<br>1: Multimedia training<br>2: Practical	Text-based training	Documentation of HBV vaccine status:<br>Intervention (E-group): 29 patients were seen by 15 residents.<br>Control: 17 patients were seen by 10 residents.<br>Residents in the E-group were not more likely to have documented the HBV vaccine status of their patients (10% vs 12%, respectively, P=1.00).

			training<br>3: Combination		
Ruf *et al.* 2010	112 general practices, 91 patients / Primary care practitioners	Problems, Affecting, Concerns, Treatment (PACT) score, OSCE	Online quality improvement program for alcohol-related disorders (online system, web-site)<br>1. Online+GP training (blended 1)<br>2. Online+GP training +team training (blended 2)	Access to the online quality improvement program for alcohol related disorders	Cross-cultural communication:<br>Students (n=119) who participated in the online module (n=60) demonstrated increased use of cross-cultural communication PACT questions compared to the control group (n=59).
Kulier *et al.* 2012	60 training units, 204 post-graduate trainees in obstetrics and gynaecology / Obstetrics and gynaecology	Questionnaire	A clinically integrated eLearning course incorporating the WHO reproductive health library for teaching basic EBM	Self-directed EBM course	Skills (OSCE scores)<br>Intervention (n=123): mean = 9.1, 95% CI: 8.7 to 9.4; Control (n=81): mean = 8.3, 95% CI: 7.9 to 8.7; Adjusted difference in mean = 0.7, 95% CI: 0.1 to 1.3; P=.02

			among postgraduates		

ATLS Advanced Trauma Life Support; CPD: continuing professional development; EMB: evidence-based medicine; MCQ: multiple choice question; OSCE: objective structured clinical examination, HBV: Hepatitis B virus, CSD: Code Status Discussions, ADR: Adverse Drug Reaction
