# Supplementary material for: Online Digital Education for Postregistration Training of Medical Doctors: Systematic Review by the Digital Health Education Collaboration
Source: J Med Internet Res. 2019 Feb 25;21(2):e13269. doi: 10.2196/13269 (PMC6410118; doi:10.2196/13269)
Supplement: Multimedia Appendix 7 [file jmir_v21i2e13269_app7.pdf]

Multimedia Appendix 7: Characteristics of included studies assessing attitude

Study ID	No. of participants / Specialty	Assessment method	ODE type	Control	Post-intervention attitude
ODE vs self-directed learning					
Connolly *et al.* 2014	56 / Paediatrics	Likert scale	'Beyond Milestones' online interactive teaching resource	Self-directed learning (text-based training)	Intervention (teaching group): n=30<br>Control: n=26<br>The teaching group reported higher confidence in their developmental assessment skills as compared to controls (MeD = 0.56; *t* (44) = −2.170; P = .035).
Harris *et al.* 2002	99 / Multispecialty	Questionnaire	Interactive case-based domestic violence education program	Self-directed learning (text-based training)	Eight elements of attitude were reported. Asking about domestic violence is presented here. Intervention (n=50): mean change = 0.91; control (n=49): mean change = 0.01, P=.08
Le *et al.* 2010	24 / Paediatrics	Likert scale	Web-based multimedia learning modules for physicians' knowledge, attitudes and treatment for paediatric asthma	Self-directed learning	Fifteen domains of attitude were measured, however no difference was found for 14 of these. We report on 'Patients with daily asthma symptoms should be prescribed an ICS'. Intervention (n=15): MD = 0.3 (SD = 0.5); control (n=9): MD= -0.2 (SD = 1.6).
Sullivan *et al.* 2010	213 / General medicine or internal medicine	Questionnaire	Web-based module on opioid therapy for chronic non-cancer pain	Self-directed learning (opioid guidelines)	Four domains of attitude were measured. The study reported mixed results for attitude. We report the results only for 'Agree to prescribe opioids when

					patients request this' in the data analysis. Intervention (COPE course; n=109): mean = 37.8 (SD=27.4); Control (VA guidelines; n=104): mean = 38 (SD=29.9).
ODE vs face-to-face learning					
Pelayo-Alvarez *et al.* 2013	169 / Primary care practitioners	Questionnaire	Palliative care education (online program)	Face-to-face (traditional palliative care training course)	Intervention: n=85 Control: n=84 Confidence in patient symptom management and confidence in communication of diagnosis and disease prognosis showed no differences between groups at 18-months.
Putnam *et al.* 2015	51 / Surgery	Questionnaire	Online curriculum on patient safety	Face-to-face learning (resident safety workshop)	Safety culture at 6-months Intervention: n=26, 68% Control: n=25, 73%
ODE vs other types of ODE					
Yardley *et al.* 2013	346 / General practitioners	Questionnaire	1. CRP group 2. Communication group 3. Combined group	Text-based training	Importance of reducing prescribing. CRP group (n=73): mean = 6.2 (SD = 1) Communication group (n=83): mean = 6.3 (SD = 0.9)
Blended learning vs self-directed / face-to-face learning					
Kulier *et al.* 2009	6 training units, 61 (pg. trainees in obstetrics and gynaecology/ Obstetrics and Gynaecology	Likert scale	eLearning course for teaching basic EBM among postgraduates	Face-to-face learning (lecture)	Seven domains of attitude were reported; we have presented results for question G. Intervention (n=15): attitude gain = 14%; attitude unchanged = 57%; and attitude loss = 29%. Control (n=25): attitude gain = 41%; attitude unchanged = 45%; and attitude loss = 14%.

COPE: Collaborative Opioid Prescribing Education; EMB: evidence-based medicine, CRP: C-Reactive Protein test
