# Supplementary material for: Online Digital Education for Postregistration Training of Medical Doctors: Systematic Review by the Digital Health Education Collaboration
Source: J Med Internet Res. 2019 Feb 25;21(2):e13269. doi: 10.2196/13269 (PMC6410118; doi:10.2196/13269)
Supplement: Multimedia Appendix 8 [file jmir_v21i2e13269_app8.pdf]

Multimedia Appendix 8: Characteristics of included studies assessing satisfaction

Study ID	No. of participants / Specialty	Assessment method	ODE type	Control	Post-intervention satisfaction
ODE vs self-directed learning					
Bell *et al.* 2000	162 / Primary care practitioners	Questionnaire	Self-study Acceleration with Graphic Evidence (SAGE) or printed materials on acute myocardial infarction	Self-directed learning (printed self-study materials)	SAGE users (n=83) were more satisfied with learning compared to those in the printed material group (n=79): median rating = 17.0 vs 15.0; P<.001.
Claxton *et al.* 2011	82 / General medicine or internal medicine	Likert scale	Fast Facts and Concept (FFAC) emails	Self-directed learning (no emails)	Intervention: n=41<br>Control: n=41<br>No difference between pre- and post-satisfaction in both the control (P=.69) and intervention groups (P=.08).
Gold *et al.* 2004	69 / Surgery	Questionnaire	Internet CD-ROM thoracic surgery eLearning system - a hybrid Prerequisite Curriculum (PRC)	Self-directed learning (curriculum outline)	Comfort, satisfaction, confidence, interest communication, study habits, organization etc. Post-matriculation resident assessment and resident performance results:<br>+PRC (n=46): mean score=3.43;

					-PRC (n=32): mean score = 2.87, P<.05. Post-matriculation faculty assessment survey and resident performance results: +PRC (n=76): Mean score = 3.84; -PRC (n=83): Mean score = 3.76; P>0.05.
Matzie *et al.* 2009	Surgical residents: 55; Fourth year-medical students: 324 / Surgery	Likert scale	Spaced education on how to provide effective feedback (emails)	Self-directed learning (text-based learning)	Students: intervention (n=149), controls (n=175). Students reported resident feedback was 'helpful' in their learning in 92% (132 of 143) of their evaluations of spaced education residents, compared to 82% (132 of 161) of their evaluations of control residents (RR= 1.13, P=.01).<br><br>Frequency of feedback RR=1.43, (95% CI: 1.08 to 1.90, P =.016.
Pernar *et*	29 / Surgery	Likert scale	Improve teaching	Self-	Group A (intervention,

al. 2012			skills in a surgery department using spaced education	directed learning	n=15) and Group B (control, n=14) 87.1% (Group A) and 89.9% (Group B) (P=.15) of faculty were felt to deliver useful feedback; 89.2% and 90.8% (P=.71) of faculty were perceived to encourage student autonomy; and 78.1% and 81.9% (P= 0.89) of faculty were felt to set clear learning expectations for students.
Sullivan *et al.* 2010	213 / General medicine or internal medicine	Questionnaire	Web-based module, opioid therapy for chronic non-cancer pain	Self-directed learning (opioid guidelines)	General satisfaction: Intervention: pre-test mean (n=109) = 56.9 (SD = 7.1); post-test mean (n=88) = 61.9 (SD = 8.6). Control: pre-test mean (n=104) = 57.4 (SD = 8.2), post-test mean (n=85) = 60.4 (SD = 7.7).
ODE vs face-to-face learning					
Bello *et al.* 2005	56 / Anaesthesiology	Questionnaire	Online teaching	Face-to-face learning	Overall satisfaction scores.

				course	Online course (n=28): median = 10 Traditional course (n=28): median = 9; P= .014.
Chenkin *et al.* 2008	21 / Emergency medicine	Likert scale	Web-based ultrasound-guided vascular access training	Face-to-face learning (didactic group)	Found overall course to be useful: Intervention (n=11): Satisfied: 100% Control (n=8): Satisfied: 80%; P=.21
Fordis *et al.* 2005	103 /Primary care practitioners	Questionnaire	1. Online CME on cholesterol management 2. Live interactive CME	Face-to-face learning (lecture)	Online CME (n= 52): Satisfaction: 94% Live CME (n=51): Satisfaction: 100%
Hemmati *et al.* 2013	80 / Primary care practitioners	Questionnaire	Cardiopulmonary resuscitation (CPR) curriculum guidelines training through Internet-based learning	Face-to-face learning (CPR lecture)	Participants in the intervention group (n=40), had significantly higher satisfaction scores (mean = 62.5; SD = 2.32) than those in the control group (n=40) (mean = 54.6; SD= 2.18).
ODE vs other types of ODE					
Schroter *et al.* 2011	1054 / Multidisciplinary	Questionnaire	Diabetes Needs Assessment Tool (DNAT)	Diabetes learning modules	15 measures of satisfaction were reported, we have only

					reported on the results for 'Learning materials have improved my overall understanding of diabetes.' Intervention group (n = 285): yes=237. Control group (n=295): yes=251.
Shaw *et al.* 2012	371 / Multispeciality	Likert scale	Online spaced education program to improve knowledge and compliance with the National Patient Safety Goal (NPSG)	Online slide show based online program	Several domains of satisfaction were measured, we have only reported on the results for 'intervention improved'. Intervention: (n=118): yes=65% Control (n=41): yes=42% P=.003
Yardley *et al.* 2013	346 clusters (GPs); 2886 patients / Primary care practitioners	Questionnaire	1. CRP group 2. Communication Group 3. Combined group	Usual care	Patients in the CRP group reported slightly lower levels of satisfaction with the consultation, *F* (3, 2315) = 4.39, P = .004. Satisfaction questionnaire scores

					were generally high, with a mean of 8.26 (SD = 1.52) out of a maximum score of 10, with no significant differences between groups or countries.
Blended learning vs self-directed / face-to-face learning					
Ali *et al.* 2013	30 / Family practice residents	Questionnaire	ATLS delivered through telemedicine	Standard ATLS course	Overall rating of the course:<br><br>Intervention (n=14): mean: 3.91 (SD=0.3)<br><br>Control (n=16): mean: 3.67 (SD=0.5) P=.20
Kronick *et al.* 2003	81 / Not specified	Questionnaire	3-hours training on using the World Wide Web to research patient related questions	Self-directed learning (text-based learning)	8 domains of satisfaction were measured, we have only reported on the results for 'comfort in using email'.<br>Intervention (n=30): mean score = 3.6 (SD = 1.3)<br>Control (n=40): mean score = 3.4 (SD = 1.3)
Platz *et al.* 2010	55 / Emergency medicine	Likert scale	Web-based basic ultra-sonographic principles and the	Face-to-face learning	Satisfaction was measured only for the Classroom and Web

			Extended Focused Assessment with Sonography for Trauma (EFAST) training		group. We have only reported on the results for scale '1=very much' in response to 'enjoyed didactic training'.<br>Web (n=21): rating = 1 (42.9%)<br>Class (n=19): rating = 1 (79.0%); P<.000

ATLS Advanced Trauma Life Support; CME: continuing medical education, CRP: C - reactive protein test
