# Supplementary material for: Online Digital Education for Postregistration Training of Medical Doctors: Systematic Review by the Digital Health Education Collaboration
Source: J Med Internet Res. 2019 Feb 25;21(2):e13269. doi: 10.2196/13269 (PMC6410118; doi:10.2196/13269)
Supplement: Multimedia Appendix 9 [file jmir_v21i2e13269_app9.pdf]

Multimedia Appendix 9: Characteristics of included studies assessing practice or behavior change

Study ID	No. of participants / Specialty	Assessment method	ODE type	Control	Post-intervention practice/ behavior change
ODE vs self-directed learning					
Bell *et al.* 2015	155 / Primary care practitioners	Clinicians' behavior was assessed with transcripts and standardized patients	Interactive web-based genetics curriculum	Self-directed learning (genetic review articles)	Intervention: n=77<br>Control: n=78<br><br>Several domains of behavioral change were measured, we report on 'Benefits of genetic counselling'.<br><br>Numbers of physicians who raised the topic: Intervention: n=47; Control: n=37
Braido *et al.* 2012	44/ Primary care practitioners	Data from a database	Respiratory allergic diseases: monitoring study of GINA and ARIA guidelines (blended CME)	Self-directed learning	Mean percentage difference between the training and control groups found a significant increase in spirometry in the training group (+63.4%, P<.01) and a non-significant increase in RASTs

					(+33.6%), prick tests (+31.7%), spirometry with bronchodilation (+46.2%), and methacholine tests (+66.5%).
Butler *et al.* 2012	68 clusters (general practice), 263 clinicians / Primary care practitioners	Hospital chart audit	Stemming the Tide of Antibiotic Resistance (STAR) educational program	Self-directed learning (text-based training)	Antibiotic dispensing rate /1000 registered patients.<br>Intervention (n=139): baseline mean = 678; follow-up mean: 664.<br>Control (n=124) clinicians, baseline mean: 669, follow-up mean: 681<br>% reduction in intervention relative to control: 4.2, 95% CI: 0.6 to 7.7, P=.02
Curtis *et al.* 2007	153 / Primary care practitioners	Hospital chart audit	Web-based glucocorticoid-induced osteoporosis (GIOP) course (three case-based scenarios)	Self-directed learning (text-based training)	BMD testing rates:<br>Intervention (n=78): 19%<br>Control (n=75): 21%<br>Rate difference: −2%, 95% CI: −8% to 4%, P=0.48

					Any osteoporosis medication prescribing: Intervention (n=78): 32% Control (n=75): 29% Rate difference: 3%, 95% CI: −3% to 9%, P=.34
Dayton *et al.* 2000	29 / General medicine or Internal medicine	Scenario based decision support system	Computerised decision-support system for applying the ATS/CDC guidelines (Group A)	Self-directed learning (guideline card)	Concordance with ATS recommendations: Intervention (n=12): 96% Control (n=17): 57%, P<.001
Farah *et al.* 2012	45 / General medicine / Internal medicine	MCQs	Information aids, decision aids and the Internet (prostate cancer screening)	Self-directed learning (information aid, delayed intervention)	Doctors in the intervention group were more likely to appreciate PSA screening: 91% versus 60%, P=.02. Doctors in the intervention group were also more knowledgeable in recognising that PSA/DRE testing is

					associated with potential harm: 65% versus 91%, P=.04. Doctors in the intervention group were better able to correctly identify current literature findings regarding prostate cancer screening: 35% versus 75%, P=.03.
Feng *et al.* 2013	118/ Primary care practitioners	Questionnaire	Interactive web-based curriculum for prostate cancer screening 1.Intervention A: standardized patient 2. Intervention B: similar patient focus, web-based tool. (blended learning)	Self-directed learning (brochures)	Intervention: n=61 Control: n=57 Intervention physicians showed somewhat more shared decision-making behaviors (intervention 4 items vs control 11 items, P <.05), were more likely to mention no screening as an option, intervention 63% vs control (n=57) 26%, P <.05), to encourage patients to consider

					different screening options (intervention 62% vs control 39%, P <.05) and seeking input from others (intervention 25% vs control 7%, P <.05).
Gerbert *et al.* 2002	71/ General medicine or Internal medicine	Not stated	Skin cancer triage tutorial	Self-directed learning (delayed intervention)	Intervention group scored significantly higher than the control group in nine of the 14 outcome measures; this improvement was maintained for five of the nine outcomes.
Le *et al.* 2010	24 / Paediatrics	Likert scale	Web-based multimedia learning modules for physicians' knowledge, attitudes and treatment of paediatric asthma	Self-directed learning	This study presents two behavioral outcomes, we report only on 'considering the total number of your patients with persistent asthma, for what percentage of these patients did you prescribe an ICS'. Intervention (n=15): mean = 6.9 (SD = 15.3).

					Control (n=9): mean = 10.5 (SD = 15.7).
Little *et al.* 2013	246 clusters (primary care practices), 4264 patients / Primary care practitioners	Case report form	1. C-reactive protein (CRP) training<br>2. Enhanced communication training<br>3. Combined training (CRP training + enhanced communication training)	Self-directed learning	Antibiotic prescription rates (crude %):<br>No CRP training: 48% (984/2040)<br>CRP training: 33% (734/2224)<br>No communication training: 45% (876/1932)<br>Communication training: 36% (842/2332).
Meeker *et al.* 2016	47 Primary care practices (248 clinicians enrolled)/ Primary care practitioners	Questionnaire	Intervention group (peer comparison) received email together with feedback and suggestions	Self-directed learning (text-based training)	Antibiotic prescribing rates for antibiotic-inappropriate acute respiratory infections:<br>Peer comparison (n= 20): mean = 19.2, 95% CI: 17.3 to 21.1<br>Control (n=27): mean = 24.0, 95% CI: 22.1 to 25.8<br>Suggested alternative (n=42): mean: 30.2, 95% CI: 28.4 to 32.1<br>Accountable

					justification (n=35): mean = 16.4, 95% CI: 14.7 to 18.0.
Short *et al.* 2006	81 / Multispeciality	Questionnaire	Online intimate partner violence (IPV) CME program	Self-directed learning	Online CME: n=44<br>Control: n=37<br>Online CME program was associated with a significant improvement in eight of<br>10 KABB outcomes, including physician self-efficacy and reported IPV management practices, over the study period.
Stewart *et al.* 2005	58 / Primary care practitioners	Clinical vignettes	Case-based on-line learning group	Wait-listed control group	The study reported knowledge on prevention and diabetes topics; we report only the mean change in quality of practice on the prevention topic at 2-months.<br>Intervention (n=27): mean = 52.2 (SD =

					11.7)<br>Control (n=31): mean = 47.7 (SD = 13.8)
Xiao *et al.* 2007	50 / Surgery, emergency medicine	Video review	Online training course on central venous catheter insertions (video group)	1. Paper group<br>2. Self-directed learning	Compliance for sterile practice is reported.<br>Video group: cases n=19<br>Paper group: cases n=31<br>Control group: cases n=23<br>The full compliance rate in the video group (14 of 19, 74%) was significantly higher (P=0.003)<br>than that in the paper and control groups (18 of 54, 33%), with an OR of 6.1 (95% CI: 1.96 to 22.03).
ODE vs face-to-face learning					
Fordis *et al.* 2005	103 / Primary care practitioners	Hospital chart audit	Intervention 1: Online CME on cholesterol management<br>Intervention 2: Live interactive	Lecture	Appropriate screening for dyslipidaemia:<br>Online CME (n=20): mean difference = -0.1, 95% CI: -2.9 to 2.6

			CME		Live CME (n = 20): mean difference = -3.3, 95% CI: -5.9 to -0.7<br>Lecture group (n=20: mean difference = -0.8, 95% CI: -3.5 to 1.8<br>Patients appropriately treated:<br>Online CME group (n=17): mean difference = 5.0, 95% CI: 1.0 to 9.1<br>Live CME group (n=19): mean difference= -1.1, 95% CI: -4.9 to 2.7<br>Control group (n=18): mean difference = 1.2, 95% CI: -2.8 to 5.1
ODE vs other types of ODE					
Allison *et al.* 2005	209 / Primary care practitioners	Data from a database	Intervention: Multicomponent Internet CME	Internet-based CME	Pre-intervention screening rates were 18.9% for the intervention (n=103) and 16.2% for the comparison offices

					(n=106) (P=.135). Post-intervention screening rates were 15.5% for the intervention and 12.4% for the comparison offices (P=.044).
Schroter *et al.* 2011	1054 / Multidisciplinary	Questionnaire	Diabetes Needs Assessment Tool (DNAT)	Diabetes learning modules	Three practice changes were measured; we report on 'Awareness of change in level of competence'. Intervention (n=291): yes = 217 (74.6%) Control (n=306): yes = 229 (74.8%) Difference: -11.3, 95% CI: -17.8 to -4.8
Shaw *et al.* 2012	371 / Multispeciality	Questionnaire	Online spaced education program to improve knowledge and compliance with the National Patient Safety Goal (NPSG)	Slide show based online program (SQ) to improve knowledge and compliance with the National	Spaced education (SE) interns demonstrated a mean of 4.79 (36.6%) NPSG-compliant behaviors (out of 13 total), while SQ interns demonstrated a mean of 4.17 (32.0%) (P=.09).

				Patient Safety Goal (NPSG)	
Weston *et al.* 2008	113 / Primary care practitioners	Compliance assessed by reviewing video records of CVC insertion and marking score sheets.	Type 2 diabetes seminar	Systolic heart failure seminar	The study reported intentions to change patient management on a 3-point scale (yes, no and not sure). We report on responses for option 'yes'. Diabetes group (n=64): yes=19 (31.2%) Systolic heart failure group (n=49): yes=13 (28.9%)
Blended learning vs self-directed learning/face-to-face learning					
Daetwyler *et al.* 2010	54 / General or Internal medicine	Checklist	Drexel University College of Medicine's (DUCOM) group's "doc.com" and web objective structured	Self-directed learning	Number of correct behaviors scored by standardized patients: "Doc.com + WebOSCE" (n=16): baseline mean = 44%, (SD = 21%), final mean = 71% (SD = 12%), mean change =

			clinical exam "WebOSCE" Intervention 1: DUCOM+MCQ Intervention 2: DUCOM +MCQ+ second web encounter exercise		27% (SD = 21%) Control group (n=19): baseline mean = 56% (SD = 20%), final mean = 63% (SD = 14%), mean change = 8% (SD = 27%)
Epstein *et al.* 2011	8 clusters (practices), 49 paediatricians / Paediatrics	Hospital chart audit	Internet portal to improve community-based paediatric ADHD Care	Delayed intervention (6-months)	Several behavioral change outcomes were reported; we report only on the 'use of parent rating of ADHD during assessment at 6-months'. Intervention (n=27): mean = 42 (SD = 25.9) Control (n=22): mean: 18.1 (SD = 33.1).
Ruf *et al.* 2010	112 clusters (general practices), 91patients / Primary care practitioners	Questionnaire	Online improvement program for alcohol-related disorders (online system, web-site)	Control: Access to the online system without any training	Patient documentation at follow-up, ITT analysis. GP group (n=43): yes = 7 GP+Nurse group

			1. Online+GP training<br>2. Online+GP training +team training		(n=42): yes = 8<br>Control (n=27): yes = 4<br>Test for difference between groups, exact test=0.26, P=.90.
Midmer *et al.* 2006	88 / Primary care practitioners	Hospital chart audit	Opioid- and benzodiazepine-prescribing skills (emails) + Discussions	Face-to-face learning (3-hr interactive presentation)	No significant before-after changes on self-reported behaviors or on clinical confidence comfort levels, motivation, or rating of importance.

ARIA: Allergic Rhinitis and its Impact on Asthma; ATS: American Thoracic Society; CDC: Centers for Disease Control; CME: continuing medical education; GINA: Global Initiatives for Asthma guidelines; MCQ: multiple choice question, KABB: Knowledge, Attitudes, Beliefs, and Self-reported behaviors, CVC: Central Venous Catheter, SE: Spaced Education, SQ: Slide-show based online program, PSA: Prostate-Specific Antigen, DRE: Digital Rectal Examination, BMD: Bone mineral density, RASTs: Radioallergosorbent tests
