# Supplementary material for: Online Digital Education for Postregistration Training of Medical Doctors: Systematic Review by the Digital Health Education Collaboration
Source: J Med Internet Res. 2019 Feb 25;21(2):e13269. doi: 10.2196/13269 (PMC6410118; doi:10.2196/13269)
Supplement: Multimedia Appendix 10 [file jmir_v21i2e13269_app10.pdf]

Multimedia Appendix 10: Characteristics of included studies assessing patient outcomes

Study ID	No. of participants / Specialty	Assessment method	ODE type	Control	Post-intervention patient outcomes
ODE vs self-directed learning					
Butler *et al.* 2012	68 clusters (general practices), 263 clinicians / Primary care practitioners	Hospital chart audit	Stemming the Tide of Antibiotic Resistance (STAR) educational program	Self-directed learning (text-based training)	Hospital admissions: There were no significant differences in admissions between the groups. Re-consultation rates: There were no significant differences between intervention and control groups for re-consultation rates after an index consultation, expressed as re-consultations per 1000 registered patients, for respiratory tract infections the median difference was (intervention−control) −0.65 (−1.69 to 0.55) at seven days; −1.33 (−2.12 to 0.74) at 14 days; and −2.32 (−4.76 to 1.95) at 31 days.
Dolan *et al.* 2015	41 / General medicine or internal medicine, 458	Hospital Chart audit	Online formative self-assessment curriculum in bone health	Self-directed learning (text-based learning)	Proportion of female patients appropriately screened for osteoporosis: Intervention: patient

	patients				screened = 227; appropriately screened: 216 (95.2%)<br>Control patients = 231; appropriately screened = 206 (89.2%), P=.02
ODE vs other types of ODE					
Estrada *et al.* 2011	205 practices with 95 physicians and their 1182 patients / Multispeciality	Hospital chart audit	Multi-component interactive intervention including web-based CME, performance feedback and quality improvement tools	Online web resource for diabetes without feedback or electronic communication	Intervention: n=102 practices<br>Control: n=103 practices<br>The proportion of patients with A1c <= 9% was similar at baseline and follow-up in both the control (AOR = 0.94; 95% CI: 0.61 to 1.47) and intervention arms (AOR = 1.16 (95% CI: 0.80 to 1.69)<br><br>BP <140/90 mm Hg and LDL <130 mg/dl were also similar at both measurement points (P=.66, P=.46; respectively).
Franchi *et al.* 2016	697 / Geriatrics and internal medicine	Not stated	Web-based training including notions of CGA and geriatric pharmacology,	eLearning refresher on the basic notions of geriatric pharmacology	Potentially inappropriate medication (PIM) among patients:<br>Intervention (n=347): PIM = 155<br>Control (n=350): PIM =

			together with training for the use of a third generation assessment instrument (InterRAI Acute Care)		137<br>OR = 1.29, 95% CI: 0.87 to 1.91, P=0.2<br>At least one potential drug-drug-interaction (DDI):<br>Intervention (n=347): DDI = 297<br>Control (n=350): DDI = 320<br>OR = 0.67, 95% CI: 0.34 to 1.28, P= .2
Kerfoot *et al.* 2014	111 / Primary care practitioners	Questionnaire	Spaced education for hypertension management	Identical educational content in an online posting	The study reported on many patient-related outcomes; we report on the 'medication possession ratio' between the spaced education game group and the control group.<br>Intervention (n=7224): mean = 0.94, (SD = 0.08)<br>Control (n=7112): mean = 0.94 (SD = 0.08)
Blended learning vs self-directed learning/ face-to-face learning					
Legare *et al.* 2012	9 clusters (family practice teaching units), 449 patients, 250 physicians/ Primary care	Hospital chart audit	DECISION+2: a shared decision-making training program to reduce the overuse of	Self-directed learning	The study reported behavior changes among patients in five preferred roles in decision-making; we report only on: 'patient decides at physician level'.

	practitioners		antibiotics for acute respiratory infections		Post-intervention: Intervention (n=160): yes=16 (10%) Control (n=108): yes=9 (8.3%)
Girgis *et al.* 2009	375 / Radiation oncology	Not stated	Consultation skills training program	Face-to-face learning (delayed intervention)	The study reported six psychosocial outcomes in patients; we have only reported on anxiety scores at 1-week. Intervention (n=193): mean = 5.2 (SD = 4.2) Control (n=183): mean = 5.2 (SD 4.1) P=.183

AOR: adjusted odds ratio; CME: continuing medical education; OR: odds ratio.
