# Supplementary material for: Online Digital Education for Postregistration Training of Medical Doctors: Systematic Review by the Digital Health Education Collaboration
Source: J Med Internet Res. 2019 Feb 25;21(2):e13269. doi: 10.2196/13269 (PMC6410118; doi:10.2196/13269)
Supplement: Multimedia Appendix 11 [file jmir_v21i2e13269_app11.pdf]

Multimedia Appendix 11: ODE intervention type by participants' specialty, country of publication and intervention duration

Study ID	Specialty	Country	Topic of the ODE content or course	Duration of the intervention (average)	Time of assessment
Ali *et al.* 2013	Primary care practitioners	Canada	Advanced Trauma Life Support telemedicine course	2 days	Post-test
Allison *et al.* 2005	Primary care practitioners	USA	Multicomponent Internet CME to increase Chlamydia screening	1 year	2 years
Bell *et al.* 2000	Primary care practitioners	USA	SAGE (Self-study Acceleration with Graphic Evidence) or printed materials on acute myocardial infarction	Median: 27, (95% CI: 25 to 30)	Post-test: 4-6 months
Butzlaff *et al.* 2004	Primary care practitioners	Germany	Computerised guidelines	3 months	2.5 months; 5 months
Butler *et al.* 2012	Primary care practitioners	UK	Stemming the Tide of Antibiotic Resistance (STAR) educational program	N/A	12 months
Bell *et al.* 2015	Primary care practitioners	USA	Interactive web-based genetics curriculum	6 hours	Post-test
Braido *et al.* 2012	Primary care practitioners	Italy	Online CME to improve knowledge of ARIA and GINA guidelines	12 months	1 year
Chan *et al.* 1999	Primary care practitioners	Canada	Access to study web-based resources (URLs) on depression but without the benefit of small-group interaction	2 months	Post-test
Curtis *et al.* 2007	Primary care practitioners	USA	Web-based glucocorticoid-induced osteoporosis (GIOP)	N/A	1 year

			course		
Fordis *et al.* 2005	Primary care practitioners	USA	Interactive online CME for cholesterol management	3.8 hours (SD = 2.0)	Post-test: 12 weeks
Feng *et al.* 2013	Primary care practitioners	USA	Interactive web-based curriculum on physician communication regarding prostate cancer screening	30-minutes	Post-test
Hugenholtz *et al.* 2008	Primary care practitioners	Netherlands	Website including an eLearning module on mental health issues	Intervention: 30 minutes<br>Control: 30 minutes	Post-test
Hemmati *et al.* 2013	Primary care practitioners	Iran	Cardiopulmonary resuscitation curriculum guidelines training through internet-based learning	145 minutes (range: 125-182 minutes)<br>Face-to-face class room: 6 hours	Post-test
Houwink *et al.* 2014	Primary care practitioners	Netherlands	Genetics eLearning CPD module about oncogenetics	Intervention: 124 minutes<br>Control: 2 hours	6 months
Houwink *et al.* 2015	Primary care practitioners	Netherlands	Genetics eLearning CPD module about oncogenetics	N/A	12 months
Kutob *et al.* 2009	Primary care practitioners	USA	Skills focused Internet-based course to teach cultural competence of type 2 diabetes	1-4 weeks	Post-test
Kerfoot *et al.* 2014	Primary care practitioners	USA	Spaced education game for hypertension management	52 weeks	52 weeks
Koppe *et al.* 2016	Primary care practitioners	Australia	Web 2.0 Balint group to improve psychological medicine skills and work-related affect	2 hours per fortnight	Post-test

Legare *et al.* 2012	Primary care practitioners	Canada	DECISION+2, a shared decision-making training program to reduce the overuse of antibiotics for acute respiratory infections	4 hours	Post-test
Little *et al.* 2013	Primary care practitioners	Belgium, Spain, Wales, Poland, UK, Netherlands	Internet-based training on antibiotic prescribing rates for acute respiratory tract infection	4 months	Post-test
Lee *et al.* 2015	Primary care practitioners	USA	Online cultural competence and problem-affect-concern-treatment training	1-hour (online teaching)	Post-test
Marsh-Tootle *et al.* 2011	Primary care practitioners	USA	Web-based intervention to improve and sustain knowledge and screening of amblyopia	N/A	Short-term: 1-hour to 17 days<br>Long-term: 1.8 years
Meeker *et al.* 2016	Primary care practitioners	USA	Behavioral interventions (peer-comparison, an email-based intervention) on inappropriate antibiotic prescribing	18 months	18 months
Ngamruengphong *et al.* 2015	Primary care practitioners	USA	Didactic lecture and periodic email reminders with immediate feedback on HBV vaccination	30 minutes (didactic lecture). Monthly email reminders of the lecture content for 2 months.	Post-test: 6 months

Pelayo-Alvarez *et al.* 2013	Primary care practitioners	Spain	Online training in palliative care	Online: 96hours Face-to-face learning: 20 hours	18 months
Ruf *et al.* 2010	Primary care practitioners	Germany	An online quality improvement program for alcohol-related disorders	4 hours	3–4 months
Stewart *et al.* 2005	Primary care practitioners	Canada	Case-based online learning of evidence-based practice guidelines in type 2 diabetes prevention	4 weeks	2 months; 6 months
Vollmar *et al.* 2010	Primary care practitioners	Germany	Dementia management using a blended learning approach	Study arm A: 75 minutes Study arm B: 45 minutes	9-weeks; 4-months
Weston *et al.* 2008	Primary care practitioners	USA	Online CME on type 2 diabetes and systolic heart failure	N/A	Post-test
Yardley *et al.* 2013	Primary care practitioners	Wales, Belgium, Netherlands, Spain, Poland	Web-based intervention to reduce antibiotic prescribing for acute lower respiratory tract infections	N/A	Post-test
Midmer *et al.* 2006	Primary care practitioners	Canada	Distance learning program on opioid and benzodiazepine prescribing skills for physicians	10 weeks	4-6 months
Ali *et al.* 2013	Surgery	Canada	Advanced Trauma Life Support course	2 days	Post-test
Ferguson *et al.* 2015	Surgery	USA	Online short course on frailty	N/A	Post-test

Gold *et al.* 2004	Surgery	USA	Internet CD-ROM thoracic surgery eLearning system, a Novel Internet Hybrid Surgery Curriculum (Prerequisite Curriculum)	57.5 hours	3 months
Macrae *et al.* 2004	Surgery	Canada	Teaching critical appraisal skills with an Internet-based journal club	8 months	Post-test
Matzie *et al.* 2009	Surgery	USA	Spaced education for improving feedback that surgical residents give to medical students	9 months	Post-test
McLeod *et al.* 2010	Surgery	Canada	Internet journal club for teaching critical appraisal skills	8 months	Post-test
Pernar *et al.* 2012	Surgery	USA	Improve teaching skills in a surgery department using spaced education	9 months (weekly emails)	Post-test
Pape-Koehler *et al.* 2013	Surgery	Germany	Multimedia-based training on Internet platforms to improve surgical performance in laparoscopic cholecystectomy using a Pelvic-Trainer	2 hours	Day 2
Putnam *et al.* 2015	Surgery	USA	Online curriculum on patient safety	NA	Post-test: 6 months; 12 months
Satterwhite *et al.* 2012	Surgery	USA	A webpage on microsurgery, entitled "Microsurgery Essentials" for residency training	1 week	Post-test
Shariff *et al.* 2015	Surgery	UK	Multimedia educational tools for cognitive surgical skill acquisition in open	N/A	1 month

			laparoscopic colorectal surgery		
Schmitz *et al.* 2016	Surgery	USA	Mastering Difficult Family Conversations in Surgical Care online course	N/A	Post-test
Claxton *et al.* 2011	General medicine or internal medicine	USA	Fast Facts and Concepts weekly emails for palliative care training	32 weeks	Post-test: 1 -8 weeks
Cullinan *et al.* 2017	General medicine	Ireland	Standard Computerised Revalidation Instrument for Prescribing and Therapeutics (SCRIPT)	1- 2 hours	Post-test: 4 weeks; 12 weeks
Daetwyler *et al.* 2010	General medicine or internal medicine	USA	Teaching bad news delivery using "doc.com" and "WebEncounter"	1 week	7-8 weeks
Dayton *et al.* 2000	General medicine or internal medicine	USA	Internet-based decision-support system for applying the ATS or CDC guidelines for tuberculosis preventive therapy	12 minutes	10 months
Dolan *et al.* 2015	General medicine or internal medicine	USA	Online curriculum in bone health	3-6 months	Post-test
Farah *et al.* 2012	General medicine or internal medicine	Australia	Information aids, decision aids and the Internet to improve doctors' knowledge on prostate-specific antigen or digital rectal examination screening and testing	4.04 hours	Post-test
Franchi *et al.*	General	Italy	Interactive online eLearning in	1 month	12 months

2016	medicine or internal medicine		order to improve drug prescription		
Gerbert *et al.* 2002	General medicine or internal medicine	USA	Internet-based Skin Cancer Triage skills tutorial	N/A	Post-test: 8-weeks
Grover *et al.* 2010	General medicine or internal medicine	USA	Arterial and central line placement with a web-based curriculum	N/A	Post-test
Sullivan *et al.* 2010	General medicine or internal medicine	USA	Web-based module on opioid therapy for chronic non-cancer pain	6 months Attitude: 45-days Satisfaction: 60-days	Post-test
Szmuilowicz *et al.* 2012	General medicine or internal medicine	USA	Internet-based multimodality communication skills (code status discussions [CSD]) intervention	6-months (2-hours per seminar, 2-hour CSD skills booster session)	2 months
Saxon *et al.* 2015	General medicine or internal medicine	USA	Hyperlink-embedded journal articles	N/A	Post-test
Wilkinson *et al.* 2016	General medicine or internal medicine	Canada	Technology-driven simulation-based cardiac ultrasonography teaching	4 hours	Post-test: 1 month
Bernstein *et al.* 2013	Paediatrics	USA	Training in Bright Futures and oral health concepts	3-6 months	3 months

Connolly *et al.* 2014	Paediatrics	Australia	'Beyond Milestones': A innovative digital resource for observation of normal child development	5 hours (intervention)	Post-test to 1 month
Epstein *et al.* 2011	Paediatrics	USA	Internet portal to improve community-based paediatric ADHD care	6 hours	6 months; 15- months
Hymowitz *et al.* 2007	Paediatrics	USA	Solutions for smoking for paediatric residency training	4 years	2 years; 4 years
Le *et al.* 2010	Paediatrics	USA	Distributed Asthma Learning Initiative (DALI) program on the role of inhaled corticosteroids in asthma management	79 minutes	Short-term follow-up: 1 - 4 months Long-term follow-up: 6 - 8 months
Sangvai *et al.* 2012	Paediatrics	USA	Injury prevention web-based modules: motor vehicle safety, bicycle safety, poison prevention, fire or burn prevention, and firearm safety	N/A	7 months
Talib *et al.* 2010	Paediatrics	USA	Preventive oral health: hands on training and web-based training on oral health counselling	30 minutes (online)	Post-test
Chenkin *et al.* 2008	Emergency medicine	Canada	Ultrasound-guided vascular access training	1 hour	2 weeks
Chung *et al.* 2004	Emergency medicine	USA	Educational website for teaching physicians about bio-terrorism	70 minutes	1 month; 6 months
Platz *et al.* 2010	Emergency medicine	Germany	Basic ultra-sonographic principles and the Extended Focused Assessment with Sonography for Trauma	Classroom: 1 day Web group: 1 or 2 days	Post-test: 8 weeks

			(EFAST)		
Barthelemy *et al.* 2017	Emergency medicine	France	Online Modular Object-Oriented Dynamic Learning Environment (Moodle) for ECG interpretation	4 months	Post-test
Kerfoot *et al.* 2007	Urology	USA, Canada	Online educational program on the spacing effect principle for the acquisition and retention of medical knowledge	27 weeks	6 months
Bello *et al.* 2005	Anaesthesiology	Italy	Online vs live methods for teaching principles and practice of difficult airway management	36 hours	48 hours after completion
Sharma *et al.* 2013	Anaesthesiology	UK	Internet and simulation- based training on transesophageal echocardiography in anaesthetic trainees	90 minutes	Post-test: 3 weeks
Edrich *et al.* 2016	Anaesthesiology	USA	Web-based training of lung ultrasound for the exclusion of pneumothorax	30 minutes	24-hours; 4 weeks
Girgis *et al.* 2009	Radiation oncology	Australia	Consultation skills training program for oncologists	2 days	1-week; 3 months
Alfieri *et al.* 2012	Radiation oncology	Canada	Web-based radiation oncology module	2.02 hours	Post-test
Enders *et al.* 2006	Public health	USA	Internet-based introductory biostatistics course	80 hours	Post-test
Hearty *et al.* 2013	Orthopaedics	USA	ELearning for orthopaedic resident preparedness for closed reduction and pinning of paediatric supracondylar fractures	N/A	Post-test
Viguier *et al.*	Orthopaedics	France	Online training on skin	3 weeks	3 weeks

2015			tumours		
Kulier *et al.* 2009	Obstetrics and gynaecology	Netherlands, UK	Clinically integrated eLearning course in evidence-based medicine	4-6 weeks	Post-test
Kulier *et al.* 2012	Obstetrics and gynecology	Argentina, Brazil, Democratic Republic of the Congo, India, Philippines, South Africa, Thailand	Clinically integrated eLearning course in evidence-based medicine for reproductive health training	8 weeks	4 weeks
Cabrera-Muffly *et al.* 2015	Otolaryngology (ENT)	USA	Online otolaryngology module	1 year	N/A
Conroy *et al.* 2015	Pharmacology	UK	Liverpool adverse drug reaction (ADR) Causality Assessment eLearning package	1 hour	Post-test
Thompson *et al.* 2012	Gastroenterology	USA	Web-based intervention to improve knowledge of quality performance measures associated with endoscopy among gastroenterology trainees	N/A	6 weeks
Wang *et al.* 2013	Radiology	USA	Computer-based interactive simulation for teaching contrast reaction management	Intervention: 5 hours	Post-test: 4-months

			to radiology trainees		
Chang *et al.* 2014	Multi-speciality (emergency medicine, paediatrics and family medicine)	USA	Asynchronous eLearning curriculum for paediatric emergency medicine training	3 hours	Post-test
Estrada *et al.* 2011	Multi-speciality (Family, general and internal medicine physicians)	USA	A web-based multi-component diabetes intervention for physicians	64.7 hours (median)	Post-test
Gyorki *et al.* 2013	Multi-speciality (general surgery, medical oncology and radiation oncology)	Australia	Improving the impact of didactic resident training with an online spaced education program on breast cancer therapy	N/A	3 months
Harris *et al.* 2002	Multispeciality (emergency medicine, general medicine, surgery, cardiology, psychiatry and orthopaedics)	USA	Internet-based education to improve physician confidence in dealing with domestic violence	2 weeks	6 weeks
Perkins *et al.* 2012	Multi-speciality (critical care or anaesthesia)	UK and Australia	Blended approach to advanced life support training (ALS) compared to conventional instructor-led ALS training	ODE: 158 minutes + 1 day (10-hour) face-to-face learning	3-26 months

Short *et al.* 2006	Multi-speciality (community physicians in the specialties of internal medicine, family medicine, medicine, paediatrics, obstetrics and gynaecology, and psychiatry)	USA	Online intimate partner violence CME program	6-8 months	6-9 months
Westmoreland *et al.* 2010	Multi-speciality (postgraduate year 1 residents from the medicine and medicine–paediatrics residency training programs from two academic years)	USA	Web-based training in geriatrics for medical residents	2.5 sessions	Post-test
Xiao *et al.* 2007	Multi-speciality (surgical and emergency medicine residents rotating through the	USA	Online training course on central venous catheter insertions on compliance with sterile practice	Video group: 19 minutes (median)	Post-test

	trauma services)				
Shaw *et al.* 2012	Multi-speciality (included learners from surgical specialties (surgery and OB-GYN) and medical specialties (medicine, anaesthesiology, emergency medicine and psychiatry programs)	USA	Online spaced education program to improve knowledge and compliance with the National Patient Safety Goal (NPSG)	4-6 weeks	4-6 weeks
Schroter *et al.* 2011	Multidisciplinary (doctors, nurses)	Wales and Germany	Online interactive diabetes needs assessment tool (DNAT) vs online self-directed learning of diabetes guidelines	4 months	Post-test
Hadley *et al.* 2010	Not specified	UK	eLearning course in evidence-based medicine for foundation (internship) training	6 weeks	Post-test
Kronick *et al.* 2003	Not specified	USA	Use of World Wide Web (online medical resources) to research patient-related questions for rural physicians	6 months	Post-test

ATS: American Thoracic Society; CDC: Centers for Disease Control.
