# Supplementary material for: Online Digital Education for Postregistration Training of Medical Doctors: Systematic Review by the Digital Health Education Collaboration
Source: J Med Internet Res. 2019 Feb 25;21(2):e13269. doi: 10.2196/13269 (PMC6410118; doi:10.2196/13269)
Supplement: Multimedia Appendix 12 [file jmir_v21i2e13269_app12.pdf]

Multimedia Appendix 12: Risk of Bias

The groups compared in the included studies were similar in all respects except in their use of the ODE technology (mode of information delivery). However, as presented in the risk of bias summary (Appendix VI) and in the risk of bias graph (Figure 5), it was often difficult to assess the risk of bias in the included studies due to the under-reporting of relevant information. Six studies were rated as having a high risk of bias for random sequence generation; one study was rated as having a high risk of bias for allocation concealment; 31 studies had a high risk of attrition bias; three studies were at high risk of reporting bias; and 25 studies were rated as having a high risk of other potential sources of bias.

Allocation (selection bias)

Random sequence generation

Six studies were rated as having a high risk of selection bias. Allison *et al.* [109], Fordis *et al.* [51] and Houwink *et al.* [33] used a pseudo-random number generator to randomise participants; in Platz *et al.* [59] participants were randomised alphabetically by surname; in Le *et al.* [76] matched pairs were assigned to the intervention and control groups; and in Xiao *et al.* [90] participants were randomised based on the month of their rotation starting time.

Forty-six studies were rated as having an unclear risk of bias as insufficient information was reported to permit judgement, and 41 studies were rated as having a low risk of bias for random sequence generation, as these studies explicitly stated the methods used for

randomisation (e.g. coin toss, computer-generated random sequence, random number table, random permuted blocks).

Allocation concealment

Platz *et al.* [59] was rated as having a high risk of bias for allocation concealment: the allocation protocol was predictable by the personnel responsible for determining the eligibility of participants and group assignment. Seventy-eight studies were rated as having an unclear risk of allocation concealment as insufficient information was reported to permit judgement. The risk of bias for allocation concealment was low in 14 studies as these studies explicitly stated the methods used for allocation concealment, e.g. centralised randomisation, the use of sealed opaque envelopes to conceal the randomisation sequence.

Blinding (performance and detection bias)

It was not expected that participants would be blinded to intervention allocation given the nature of the interventions precludes this type of blinding. However, efforts were made to blind outcome assessors in 18 of the included studies, which were assessed as having a low risk of detection bias. Epstein *et al.* [25] had a high risk of detection bias as the chart reviewers were not blinded to the treatment condition. The remaining 74 studies were judged as having an unclear risk of bias for the blinding of outcome assessment.

Incomplete outcome data (attrition bias)

Thirty-one studies were rated as having a high risk of attrition bias due to a high drop-out rate. Ten studies had an unclear risk of attrition bias as they reported insufficient information to permit judgement, and fifty two studies were rated as having a low risk of attrition bias for incomplete outcome data, as they had no missing outcome data.

Selective reporting (reporting bias)

Three studies had a high risk of reporting bias: secondary outcomes were not reported in Estrada *et al.* [23]; Ngamruengphong *et al.* [24] did not report control data for post-test comparisons; and Daetwyler *et al.* [22] did not report data on learners' understanding of the module. Twelve studies had an unclear risk of bias as insufficient information was reported to permit judgement and 78 studies were rated as having a low risk of bias for selective reporting as they reported on all prespecified outcomes of interest.

Other potential sources of bias

Other biases were assessed by examining whether significant baseline differences existed between participants in the intervention and control groups. Twenty-five studies were judged as being at high risk of 'other biases' as a result. For most of the included studies, it was difficult to assess whether the inappropriate administration of an intervention had occurred, and hence these studies were judged to have an unclear risk of 'other bias'.
