# Supplementary material for: Online Digital Education for Postregistration Training of Medical Doctors: Systematic Review by the Digital Health Education Collaboration
Source: J Med Internet Res. 2019 Feb 25;21(2):e13269. doi: 10.2196/13269 (PMC6410118; doi:10.2196/13269)
Supplement: Multimedia Appendix 13 [file jmir_v21i2e13269_app13.pdf]

Multimedia Appendix 13: Acronyms and Definitions

CENTRAL: Cochrane Central Register of Controlled Trials

CINAHL: Cumulative Index to Nursing and Allied Health Literature

CME: Is defined as 'all educational activities which serve to maintain, develop, or increase the knowledge, skills, and professional performance and relationships that a physician used to provide services for patients, the public, or the profession',[11](pg1) and CPD as 'a range of learning activities through which medical professionals maintained and developed throughout their career to ensure that they retain their capacity to practice safely, effectively and legally within their evolving scope of practice'.[12](pg4)

ERIC: Educational Resource Information Centre

Face-to-face learning: refers to classroom didactic lecture-based learning

LAN- local area network

ODE: Online Digital Education

Online and LAN-based ODE: is defined as a learning activity that uses the Internet or an intranet as a standard for participants' learning activities. These can also be referred to as 'online', 'web-based' or 'networked' interventions. In the absence of a network connection, a loss of both functionality and usability would occur to such an extent that the original intended purpose would no longer be provided and the user interaction would end. In this review we referred to both 'online' and 'LAN-based digital education' as ODE for ease of reading.

Patient outcome: is defined as any benefit derived from the intervention by patients.

Physicians' post-intervention attitudes: is defined as a learners' stance towards the intervention, patients and/or new clinical knowledge or skills or the tendency to respond positively or negatively towards new knowledge or skills acquired as a result of the intervention, measured using any validated or non-validated instrument.

Physicians' post-intervention cognitive skills: is defined as a learners' ability and capacity to adaptively carry out complex activities (technical, cognitive or interpersonal tasks) in an educational setting, measured with any validated or non-validated instrument (e.g. pre- and post-test scores, time taken to perform a procedure, number of errors made while performing a procedure).

Physicians' post-intervention knowledge: is defined as the evaluation of learners' factual gain or conceptual understanding, measured using any validated or non-validated instrument to measure differences in pre- and post-test scores, or post-test scores only if no pre-test scores were reported. If several post-test results were available, we used the difference between the pre-test and the first post-test.

Physicians' post-intervention practice or behavior change: is defined as any change in the way a physician practices or changes in physicians' behavior in diagnosing, prescribing and counselling after the intervention. We included studies that assessed satisfaction using any validated or non-validated instrument.

Physicians' post-intervention satisfaction: is defined as the level of approval of the online and LAN-based digital health educational intervention and its perceived performance

compared with their expectations of the intervention, measured using validated or non-validated instrument.

Self-directed learning: is defined as a process in which students are responsible for organizing and managing their own learning activities and needs through the available digital resources, it included studies in which there is no intervention or text based learning.

SMD: Standardized mean difference

Traditional learning: Traditional learning in post-registration medical doctors' education includes formal and informal training (self-study), thus traditional learning refers to both formal training (face-to-face didactic classroom training) and no training (self-directed training through books and journals).
